# Discovery and Validation of New Potential Biomarkers for Early Detection of Colon Cancer

**DOI:** 10.1371/journal.pone.0106748

**Published:** 2014-09-12

**Authors:** Xavier Solé, Marta Crous-Bou, David Cordero, David Olivares, Elisabet Guinó, Rebeca Sanz-Pamplona, Francisco Rodriguez-Moranta, Xavier Sanjuan, Javier de Oca, Ramon Salazar, Victor Moreno

**Affiliations:** 1 Unit of Biomarkers and Susceptibility, Cancer Prevention and Control Program, Catalan Institute of Oncology (ICO) and CIBERESP, Hospitalet de Llobregat, Barcelona, Spain; 2 Colorectal Cancer Group, Bellvitge Biomedical Research Institute (IDIBELL), Hospitalet de Llobregat, Barcelona, Spain; 3 Department of Gastroenterology, University Hospital of Bellvitge, Hospitalet de Llobregat, Barcelona, Spain; 4 Department of Pathology, University Hospital of Bellvitge, Hospitalet de Llobregat, Barcelona, Spain; 5 Department of Clinical Sciences, Faculty of Medicine, University of Barcelona (UB), Barcelona, Spain; 6 Department of General and Digestive Surgery, Colorectal Unit, University Hospital of Bellvitge, Hospitalet de Llobregat, Barcelona, Spain; 7 Department of Medical Oncology, Catalan Institute of Oncology (ICO), Hospitalet de Llobregat, Barcelona, Spain; Centro Nacional de Investigaciones Oncológicas (CNIO), Spain

## Abstract

**Background:**

Accurate detection of characteristic proteins secreted by colon cancer tumor cells in biological fluids could serve as a biomarker for the disease. The aim of the present study was to identify and validate new serum biomarkers and demonstrate their potential usefulness for early diagnosis of colon cancer.

**Methods:**

The study was organized in three sequential phases: 1) biomarker discovery, 2) technical and biological validation, and 3) proof of concept to test the potential clinical use of selected biomarkers. A prioritized subset of the differentially-expressed genes between tissue types (50 colon mucosa from cancer-free individuals and 100 normal-tumor pairs from colon cancer patients) was validated and further tested in a series of serum samples from 80 colon cancer cases, 23 patients with adenoma and 77 cancer-free controls.

**Results:**

In the discovery phase, 505 unique candidate biomarkers were identified, with highly significant results and high capacity to discriminate between the different tissue types. After a subsequent prioritization, all tested genes (N = 23) were successfully validated in tissue, and one of them, COL10A1, showed relevant differences in serum protein levels between controls, patients with adenoma (p = 0.0083) and colon cancer cases (p = 3.2e-6).

**Conclusion:**

We present a sequential process for the identification and further validation of biomarkers for early detection of colon cancer that identifies COL10A1 protein levels in serum as a potential diagnostic candidate to detect both adenoma lesions and tumor.

**Impact:**

The use of a cheap serum test for colon cancer screening should improve its participation rates and contribute to decrease the burden of this disease.

## Introduction

Colorectal cancer (CRC) is a leading cause of death worldwide, with over one million of new cases and half a million of deaths around the world every year [1]. Five-year relative survival rates are under 50%, but this greatly depends on the stage at the time of diagnosis [2]. No primary preventive measure has proven efficacy in reducing incidence, but early detection through population screening has been found to reduce mortality [3].

Nowadays there is debate about which test should be used for CRC screening. Until further evidence is collected, current European guidelines accept the fecal occult blood test followed by confirmatory colonoscopy, which is therapeutic when resectable adenomas are identified [4]. Most population-based screening programs are using guaiac based fecal occult blood test, which biochemically detects small traces of blood derived from bleeding lesions in feces, or fecal immunological test, which is based on immunodetection of human hemoglobin in feces. These tests have a sensitivity of 80% for CRC and 28% for adenoma >1 cm, and specificities in the range of 91 to 94% [5]. Moreover, patient compliance with stool-based assays tends to be low [6]. The use of colonoscopy as a gold standard for CRC screening is controversial. It has reported higher sensitivity (97%) and specificity (98%) for early detection of CRC, but it also has several pitfalls associated to it: increased economic cost; requirement of highly trained staff; uncomfortable bowel preparation; invasiveness; risk of morbidity and mortality attached to the procedure [7], [8]. Moreover, in countries where national colonoscopy screening is available, compliance has often been low [9].

Serum-based markers would be highly attractive for CRC screening since they are minimally invasive and could be integrated in any routine health checkup without the need of additional stool sampling, thereby increasing acceptance among patients. Current molecular biology techniques allow an easier generation of many hypotheses of candidate biomarkers for diagnosis, prognosis or therapeutic response in CRC, but the need for a proper validation has been often reported [10]–[12]. The underlying hypothesis is that tumor cells of CRC, even in its pre-invasive stages, suffer important genetic alterations that induce release of characteristic proteins or nucleic acids potentially detectable in biological fluids obtained by non-invasive methods such as blood or feces [11]. Detection by molecular biology techniques of these substances will serve as a biomarker of disease to develop diagnostic tests with improved predictive power of current screening tests. Therefore, the aim of the present study was to identify and validate new serum biomarkers and demonstrate their potential usefulness for early diagnosis of colon cancer.

## Methods

The biomarker assessment in this study was organized in sequential and consecutive phases for discovery, technical and biological validation, and proof of concept to test the potential clinical use of selected biomarkers. Firstly, gene expression microarray data were analyzed to identify candidate biomarkers in tissue samples from colon cancer cases and cancer-free controls (*Discovery Phase*). Secondly, using an alternative technique based on quantitative real-time PCR (RT-qPCR), a selection of differentially expressed genes was validated in the same set of tissue biopsies (*Technical Validation Phase*), as well as in biopsies obtained from an independent set of patients (*Biological Validation Phase*). Finally, the potential clinical use of the most promising validated candidates was tested in serum samples from colon cancer cases, a small set of adenomas, and cancer-free controls through the detection of the corresponding secreted protein using enzyme-linked immunosorbent assay (ELISA) tests (*Proof of Concept Phase*). Reported STARD guidelines [13] have been the basis for defining our protocol.

### Patients and samples

Main characteristics of the subjects included in the present study are shown in Table 1. Colon tumor and paired adjacent (∼5–10 cm) pathologically normal mucosa tissue samples used in this study were obtained at the time of surgery from a series of cases with an incident diagnosis of colon adenocarcinoma attending the Bellvitge University Hospital (Barcelona, Spain) between January 1996 and December 2007. Included cases were selected to form a homogenous series of patients with stage II, microsatellite-stable sporadic colon cancer. All patients underwent radical surgery and did not receive chemotherapy prior to surgery. Pathologists confirmed all colon cancer diagnoses and selected fresh tissue samples from tumor and adjacent mucosa taken from the proximal resection margin. A hematoxylin-eosin staining was performed on a slide cut of the tumor specimen to guide the pathologist in selecting an area with at least 75% of tumor cells. The stage grouping followed the authoritative UICC guide “TNM Atlas, 6th Edition”. The best approximation to this classification was derived from the information collected at the time of diagnosis for each case.

**Table 1:** Main characteristics of the subjects included in the study.

	Discovery series	Validation series	
		Tissue	Serum
**Cancer-free controls**	**n = 50**	**n = 34**	**n = 77**
*Gender*			
Male	27 (54%)	16 (47%)	39 (51%)
Female	23 (46%)	18 (53%)	38 (49%)
*Median age (range in years)*	63 (25–88)	62.5 (50–69)	67 (34–89)
*Biopsy localization*			
Right colon	27 (54%)	26 (76%)	-
Left colon	23 (46%)	8 (24%)	-
**Patients with adenoma**			**n = 23**
*Gender*			
Male	-	-	16 (70%)
Female	-	-	7 (30%)
*Median age (range in years)*	-	-	60 (53–69)
*Adenoma localization*			
Right colon	-	-	4 (17.39%)
Left colon	-	-	17 (73.91%)
Rectum	-	-	2 (8.70%)
*Median size (range in mm)*	-	-	12 (4–23)
*Histological type*			
Tubular	-	-	7 (30.43%)
Tubulovillous	-	-	15 (65.22%)
Not available	-	-	1 (4.35%)
*Degree of dysplasia*			
High	-	-	8 (34.78%)
Low	-	-	14 (60.87%)
Not available	-	-	1 (4.35%)
**Cases**	**n = 100**	**n = 70**	**n = 80**
*Gender*			
Male	72 (72%)	39 (56%)	52 (65%)
Female	28 (28%)	31 (44%)	28 (35%)
*Median age (range in years)*	71.5 (43–87)	68.5 (41–91)	66 (22–83)
*Tumor localization*			
Right colon	39 (39%)	35 (50%)	19 (23.75%)
Left colon	61 (61%)	35 (50%)	37 (46.25%)
Rectum	-	-	24 (30%)
*Histological grade*			
High	6 (6%)	17 (24%)	18 (22.5%)
Low	94 (94%)	53 (76%)	60 (75%)
Not available			2 (2.5%)
*Tumor stage*			
I	-	-	9 (11.25%)
II	100 (100%)	70 (100%)	27 (33.75%)
III	-	-	34 (42.25%)
IV	-	-	10 (12.5%)
*T - Primary tumor*			
T1	-	-	9 (11.25%)
T2	-	-	9 (11.25%)
T3	92 (92%)	61 (87%)	46 (57.5%)
T4	8 (8%)	9 (13%)	16 (20%)
*N - Regional lymph nodes*			
N0	100 (100%)	70 (100%)	44 (55%)
N1	-	-	22 (27.5%)
N2	-	-	14 (17.5%)
M – Distant metastasis			
M0	100 (100%)	70 (100%)	70 (87.5%)
M1	-	-	10 (12.5%)
*Mean lymph node yield*	19.6	31	28.8
*Extramural vascular invasion*			
Present	7 (7%)	16 (22.9%)	30 (37.5%)
Absent	93 (93%)	54 (77.1%)	50 (62.5%)

Tissue samples of colon mucosa from cancer-free controls were obtained through colonoscopy between February and May 2010. A series of consecutive patients who underwent colonoscopy indicated by symptoms (usually anemia, bleeding, gastrointestinal pain or altered rhythm) were invited to participate. Those with negative results (i.e. without colonic lesions) were included in this study. None of them reported family history of cancer.

Finally, serum samples from colon cancer cases and cancer-free controls were selected from an epidemiologic case-control study on gene-environment interactions that has been previously described in detail [14]. All serum samples were collected prior to surgery for cases and just before colonoscopy for controls.

To simplify naming different sample types, here we will use *tumor* (T) when referring to tumor samples from colon cancer patients, *adjacent normal* (A) when referring to pathology normal colon mucosa samples from colon cancer patients, and *cancer free* (F) when referring to colon mucosa samples from cancer-free individuals.

The Clinical Research Ethics Committee of the Bellvitge University Hospital approved the study protocol, and all individuals provided written informed consent to participate and for genetic analyses to be done on their samples.

### RNA extraction

Total RNA was isolated from frozen tissue samples using Exiqon miRCURY RNA Isolation Kit (Exiqon A/S, Denmark), according to manufacturer's protocol, and considering all recommended precautions to avoid RNA degradation by RNases. Extracted RNA was quantified by NanoDrop ND-1000 Spectrophotometer (Nanodrop Technologies, Wilmington, DE) and stored at -80°C. The quality of these RNA samples was further checked using RNA 6000 Nano Kit (Agilent Technologies, Santa Clara, CA) following manufacturer's guidelines, and was confirmed by gel electrophoresis. RNA integrity numbers (RIN) showed good quality ([Q1 = 7.5; Median = 8.25; Q3 = 8.9] for tumors, [Q1 = 7; Median = 7.5; Q3 = 8] for adjacent normal and [Q1 = 7.8; Median = 8.3; Q3 = 8.65] for healthy normal). RNA purity was measured with the ratio of absorbance at 260 nm and 280 nm (mean = 1.96, SD = 0.04), with no differences among tissue types.

### Discovery series - expression arrays

The discovery series included 100 pairs of tumor and adjacent normal colonic mucosa samples and 50 samples of colonic mucosa from cancer-free individuals (total n = 250). Total RNA extracted from these samples was hybridized onto Affymetrix Human Genome U219 array plates (Affymetrix, Santa Clara, CA) following manufacturer's recommendations. Four samples (two adjacent normal-tumor pairs) were excluded from the dataset after quality control. Thus, a final dataset of 246 arrays was used for subsequent analyses.

Raw data were normalized using the Robust Multiarray Average algorithm [15] implemented in the *affy* package [16] of the Bioconductor suite (http://www.bioconductor.org) [17]. All statistical analysis were done with the R statistical computing software (http://www.r-project.org) [18].

Before the differential expression analysis was performed, low-variant and Y-chromosome transcripts were removed from subsequent analyses. For the remaining probesets, regularized-Student's t-tests were used to detect significant overexpression between adjacent normal (A) or tumor samples (T) and cancer-free mucosa (F). Bonferroni correction was applied to account for multiple hypothesis testing. In order to narrow down the initially obtained lists, candidate probesets were further filtered based on different criteria: low expression levels and low variability in cancer-free mucosa; large average fold change between T/F or A/F; and homogeneity of effects among multiple probes for the same gene, when available. Probesets that passed the filtering criteria were mapped to genes, the units of information used for downstream analyses.

A prioritization procedure was performed to select the best candidate genes for validation using publicly available data [19]–[24]. Criteria accounted for were related to reproducibility and specificity issues: observed reproducibility of the expression differences; very low levels of expression in blood tissue; and selection of genes with large expression in colon tissue when compared to other tissues according to GeneCards database (http://www.genecards.org) [25], though most genes were expressed in multiple tissues. The gene expression dataset is available in the National Center for Biotechnology Information's Gene Expression Omnibus [26] with GEO series accession number GSE44076 and in the project website (http://www.colonomics.org).

### Technical and biological validation – RT-qPCR for expression assessment

Expression levels of selected genes were assessed with RT-qPCR both for the discovery series and for an additional set of 104 samples (70 paired adjacent normal/tumor tissues from colon cancer patients and 34 from cancer-free controls). These samples were collected between January 1996 and June 2011 following the same protocol and stored under the same conditions as the discovery series. cDNA was synthesized from the extracted mRNA with the transcription first strand cDNA synthesis kit (Roche Applied Science, Penzberg, Germany) following standard procedures.

Two sets of primers were designed for each gene, and each set was assayed in duplicate. Three control genes were included in the assay: *ACTB*, *TPT1*, and *UBC*. *ACTB* was chosen based on the extensive previous literature pointing it as a suitable housekeeping gene for gene expression analyses in colon samples [27]–[29]. *UBC* and *TPT1* were selected based on the high stability of their expression levels across all samples in our array data (Figures S1 and S2). Interestingly, they had also been previously postulated as potentially suitable housekeeping genes for gene expression assays in colon samples [29].

Multiplexed RT-qPCRs assays were done using BioMark Dynamic Array 96×96 Plates (Fluidigm Corporation, San Francisco, CA). Resulting images were analyzed with Fluidigm Biomark software using standard parameters. Raw qPCR data were processed with the HTqPCR package v1.10.0 [30]. Before the assessment of differential expression between different tissue types, the expression matrix was filtered for quality purposes. *UBC* was finally selected as the housekeeping control based on the stability of its threshold cycle values (Figure S2). Mann-Whitney tests were used to compare expression levels between cancer-free and adjacent normal samples and between cancer-free and tumor samples. Each set of primers was analyzed independently, and the set of primers that displayed the highest significant results in the analysis of differential expression was selected as a representative.

### Identification of serum biomarkers – proof of concept for screening validity

To test the potential value for early detection, the most promising candidates from the biological validation were assayed in serum samples in a series of 80 colon cancer cases, 23 patients with adenoma and 77 cancer-free controls, all tested in duplicate to increase the precision of the experiment. Ten-milliliter samples of peripheral venous blood were collected from colon cancer cases, patients with adenomas and controls. After centrifuge for 15 minutes at 1000 rpm within 30 minutes of collection, serum was aliquoted and stored at −80°C. Commercial ELISA kits from Life Sciences Inc and R&D Systems, depending on availability, were used according to the manufacturer's instructions to assess serum protein concentrations. All assays employ quantitative sandwich enzyme immunoassay technique. The concentration of target proteins in each sample was calculated from a standard curve run in duplicate in each plate. The scientists examining these serum samples were unaware of the patient's diagnosis. A linear model adjusting for age, gender and potential batch effects was used to assess the statistical significance of the differential protein levels among groups. The association of markers with patient characteristics as age and gender, multiple epidemiological factors and tumor characteristics is shown in Table S1. Since some serum markers showed extreme values for a few subjects, a rank-based test was also performed. The results did not change in a relevant way and the p-values derived from the linear models are reported.

The number of samples used was calculated to attain a 10% precision on sensitivity and specificity estimates for expected values of 75%. This required at least 72 subjects per group. For the discovery series, this number was unbalanced to oversample tumors, which have larger variability and a wide range of candidates had to be analyzed. The validation series in serum was supplemented with a smaller subgroup of adenoma (n = 23) to explore the usefulness of the markers to detect this premalignant lesion.

## Results

### Biomarker discovery

**Figure 1:**
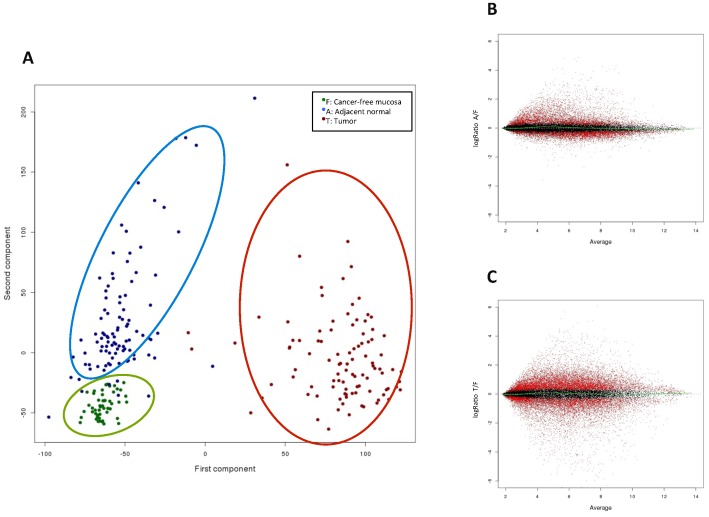
Differences in expression between tissue types in the biomarker discovery series. **A.** Principal component analysis. **B.** Differentially expressed genes between adjacent normal and cancer-free samples. **C.** Differentially expressed genes between tumor and cancer-free samples.

In this well-selected homogeneous set of samples, differences in mRNA expression measured with Affymetrix HG-U219 array plates were so remarkable that an unsupervised technique (principal components analysis) using the full set of probesets separated almost perfectly the three tissue types (Figure 1a). The first principal component clearly divides tumor samples of colon cancer cases (T) and non-tumor samples. Remarkably, the second principal component also split cancer-free (F) from adjacent normal samples belonging to patients with cancer (A).

From 33,853 probe sets included in the array with high variability, 5,503 were over-expressed in A when compared to F, and 11,229 were over-expressed in T when compared to F (p<0.05, Bonferroni corrected). We have focused specifically on over-expressed genes because these differences are more likely to be detected in serum, and therefore more suitable to be used as diagnostic biomarkers. Interestingly, a remarkable level of overlap (3,101 probe sets, ∼56%) was found between these two lists of probe sets, suggesting that adjacent normal mucosa in patients with cancer had already experienced important alterations in gene expression, and rising the importance of using cancer-free mucosa as reference tissue. Global results from differential expression analysis are shown in Figures 1b and 1c.

To prioritize candidates for diagnostic biomarkers, a set of filters were applied to the initial set of differentially expressed probe sets. These filters were based primarily on statistical criteria (i.e. large fold-change between A/F or T/F, low levels of expression and low variability in F samples). These filters yielded a final number of 242 selected probe sets between A/F and 443 between T/F, corresponding to a set of 194 and 352 genes, respectively (Table S2).

This first selection provided a list of 505 unique candidate biomarkers with highly significant expression differences between tumor and cancer-free tissue. Due to the technical difficulties derived from the validation of such a large amount of biomarkers, additional technical and biological criteria were applied to further narrow down the list of potential candidates. This second set of filters were based on assessing the consistency between the different probesets for each gene; confirmation of our results in independent and publicly available gene expression datasets; and null or low expression levels of these genes in blood samples from cancer-free individuals. Moreover, prior knowledge and molecular information for each gene was compiled from the literature and online databases to ensure the selection of the most reliable candidates (i.e. protein secretion, tissue specificity, protein function, previous evidence as a biomarker, among others). A list of the 23 best candidates was finally selected for validation in the next step (Table 2, columns 1–2).

**Table 2:** Selected genes to be technically and biologically validated.

Gene name	Type*	Discovery fold change	Discovery p-value	Technical validation p-value	Biological validation p-value
COL11A1	T/F	4.01	6.66e-36	2.06e-16	2.20e-5
KIAA1199	T/F	4.91	1.47e-71	5.94e-18	2.86e-13
MMP7	T/F	5.66	1.06e-43	4.06e-21	2.36e-15
CEL	T/F	3.14	1.83e-20	9.34e-13	6.40e-8
GAL	A/F	1.88	5.50e-29	2.65e-20	1.60e-9
MMP3	T/F	4.63	9.72e-35	2.54e-18	1.21e-12
THBS2	T/F	4.23	2.48e-38	6.71e-16	2.0e-3
COL10A1	T/F	2.67	3.08e-20	3.82e-08	5.40e-6
ESM1	T/F	2.47	2.80e-37	2.54e-18	1.05e-12
JUB	T/F	2.76	7.17e-61	2.66e-19	3.74e-08
CST1	T/F	1.92	2.88e-17	2.61e-20	2.35 e-14
MSX2	T/F	3.05	1.10e-36	2.45e-17	4.35e-9
EPHX4	T/F	2.88	5.52e-39	1.34e-20	5.61e-12
TNC	A/F	1.82	2.12e-15	1.66e-11	3.13e-4
CA9	T/F	2.62	7.75e-20	1.08e-11	4.65e-10
CLDN2	T/F	3.10	2.37e-26	9.12e-5	0.013
DPT	A/F	2.91	5.96e-36	6.01e-17	4.02e-6
SFRP2	A/F	4.02	2.29e-50	1.15e-19	2.23e-10
MMP10	T/F	1.92	7.94e-18	1.59e-13	7.65e-12
FAP	T/F	2.60	2.29e-29	1.11e-17	5.13e-12
SRPX2	T/F	3.18	3.83e-35	7.81e-12	1.57e-5
LOC100127888	T/F	1.92	2.56e-17	1.38e-09	9.55e-5
CXCL5	T/F	2.96	6.49e-19	9.6e-3	2.06e-4

*T/F: expression in tumor > expression in cancer-free mucosa; A/F: expression in adjacent normal mucosa > expression in cancer-free mucosa.

The association for all genes are significant after Bonferroni correction, but p-values shown are unadjusted for multiple comparisons.

### Technical and biological validation

To ensure the reliability of the results obtained from the gene expression arrays, the selection of 23 biomarkers was validated with an alternative technique (RT-qPCR) both in the same set of samples (i.e. technical validation), and also in an independent series with equivalent clinical and epidemiological characteristics (i.e. biological validation).

**Figure 2:**
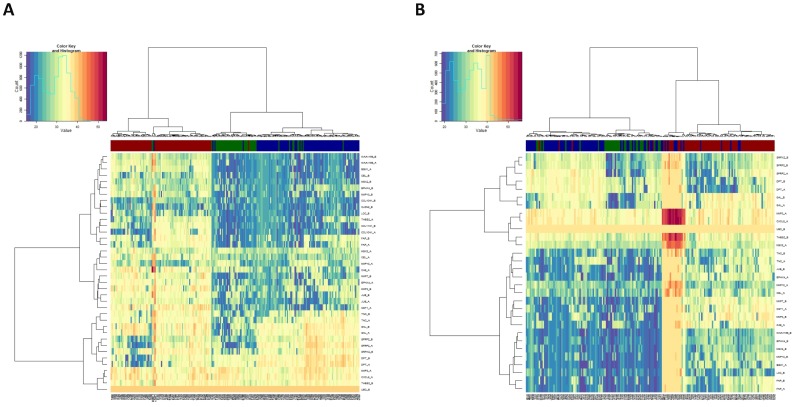
Heatmap of threshold cycle values from technical (A) and biological validation (B). Samples are color-coded on top of the heatmaps based on the tissue type (i.e., cancer-free mucosa = green, adjacent normal tissue from colon cancer patients = blue, tumor tissue = red).

Figure 2 displays two-way gene and sample clustering of the RT-qPCR expression values both for the results of the technical (Figure 2a) and the biological validation (Figure 2b). Horizontal axes of the heatmaps (i.e. columns) show a clear separation of tumor samples from adjacent normal and cancer-free groups. The vertical axis of genes (i.e. rows) showed two clusters of genes, one for those differentially expressed between A/F and another for the differentially expressed between T/F. These results highly replicate the pattern of expression observed in the arrays in the discovery phase, reinforcing the potential role of these genes as diagnostic biomarkers of colon cancer. A formal comparison of the expression differences between sample types for the technical and biological validation is shown in Table 2, columns 3–4. Although only p-values are displayed in Table 2, the expression levels of all the validated genes behaved consistently throughout the different phases, as depicted in Figure S3.

### Proof of concept – identification of serum biomarkers

As a pilot proof-of-concept to demonstrate their potential usefulness as colon cancer early diagnostic biomarkers, a selection of 9 genes were tested in serum using ELISA tests. The prioritization of these candidates was based on an extensive literature review and availability of commercial ELISA kit.

**Figure 3:**
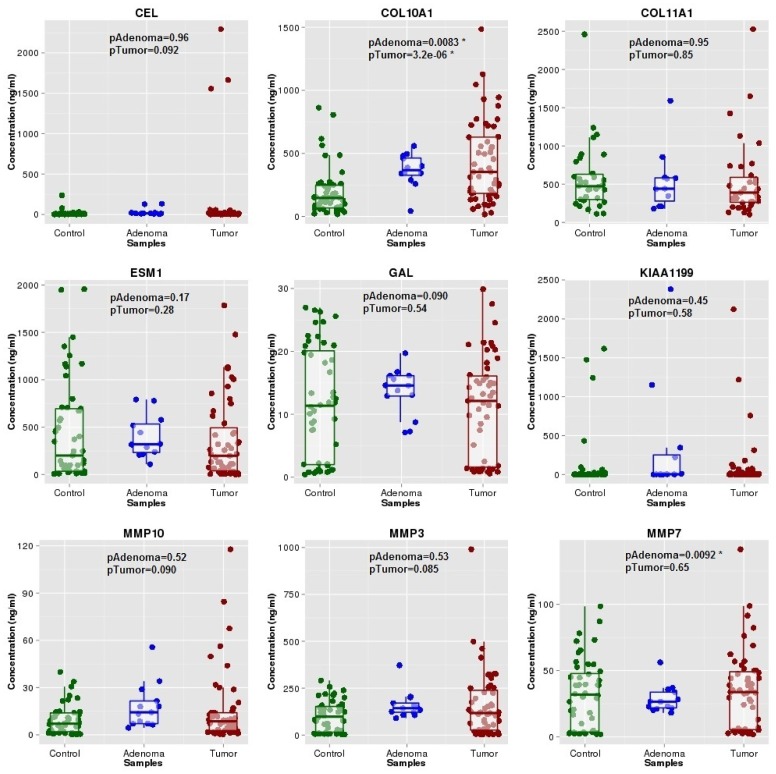
ELISA serum concentrations of each selected protein in cancer-free controls, patients with adenoma and colon cancer cases.

**Figure 4:**
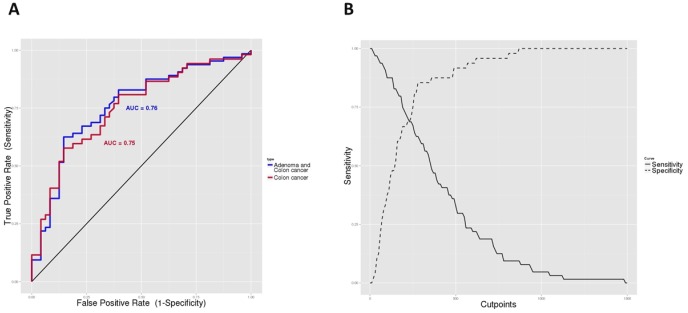
COL10A1 performance as a diagnostic biomarker. **A.** Receiver operating characteristic curves for both adenomas and colon cancer together (purple) and colon cancer cases only (red). **B.** Different marker cutpoints against the sensitivity and specificity curves.

Results for each protein are shown in Figure 3. Remarkably, collagen type X alpha1 (COL10A1) displayed very high concentrations in colon cancer cases and adenomas when compared to controls (p = 3.2e-6 and p = 0.0083, respectively). Serum concentrations of COL10A1 in controls, adenomas and colon cancer cases by stage are shown in Figure S4. Interestingly, statistically significant differences were found when controls were compared to each one of the different tumor stages, except Stage I, probably due to the small sample size of this group. The area under the receiver operating characteristic (ROC) curve was 0.75 for cancer and 0.76 when adenoma and colon cancer were considered together (Figure 4), showing potential good classification ability. Matrix metalloproteinase-7 (MMP7) also showed a significant association but was not further considered because it was due to an underexpression in adenomas compared to controls, which represented the opposite sense of differential expression that we were trying to validate. No combination of COL10A1 with any of the other proteins significantly increased the area under the ROC curve (data not shown).

## Discussion

CRC screening with fecal occult blood test has demonstrated efficacy in randomized trials. Nonetheless, the low specificity of the test suggests the need of more accurate alternative diagnostic tests. Sigmoidoscopy, colonoscopy and Computerized Tomography scan (i.e. virtual colonoscopy) are strong alternative candidates, but all have important limitations, mainly regarding costs, possible severe side effects and reduced participation. Participation is an important factor for screening effectiveness, and it is also a generalized observation that screening based on fecal occult blood test has low participation rates [31]–[33]. Thus, a diagnostic test based on a routine blood test would probably be able to reach a higher percentage of the population, and public health authorities would favor such a test if efficacy and costs were similar to fecal occult blood test. With these premises in mind, we started this study to search for diagnostic biomarkers that can be detected in blood with a simple and affordable ELISA test.

Our study of gene expression in colon tissue has confirmed previous observations that a large number of genes are deregulated in tumor when compared to adjacent normal mucosa. From about 20,000 genes interrogated in the expression array, and after filtering by several restrictive criteria, 505 unique candidate biomarkers have been identified (Table S2), with highly significant results and high capacity to discriminate between paired tumor and adjacent normal samples. A strong feature of our study design is the inclusion of a set of samples from cancer-free controls (n = 50). This has allowed us to identify genes that do not show expression differences between adjacent normal and tumor tissue from colon cancer patients, as well as to confirm that overexpressed genes in tumors do not display high expression levels in cancer-free colon tissue, which could preclude their potential use as biomarkers. We have previously described that gene expression of adjacent normal colon mucosa in a patient with cancer already has been significantly altered when compared to cancer-free colon mucosa [34], which reinforces the need of including tissue from cancer-free individuals in projects aiming to find diagnosis biomarkers for colon cancer.

The large number of candidates identified in the analysis of expression data led us to prioritize which ones were to be selected for further validation. We used a combination of criteria, which included consistency with other publicly available datasets and literature; low or no expression levels in cancer-free mucosa or other tissues; expression predominant to colon cancer tissue; and selection of secretable proteins. Since the identification of serum proteins is expensive and time consuming, we undertook a technical and biological validation of the best candidates before attempting ELISA tests. The technical validation (i.e. in the same set of samples but with a different technique) showed a remarkable reproducibility of the expression level differences measured by RT-qPCR and microarrays for all tested genes, thus confirming that the expression dataset obtained with Affymetrix HG-U219 microarrays was of outstanding quality and reliably identified expression differences between the different tissue types. Therefore, we expect that the number of false positives in the remaining list (not validated) of significant differentially expressed genes between tissue types to be low. Moreover, the confirmation of the previously identified differences in a biologically independent dataset also highlights the validity of the results obtained with microarrays.

The next step in our sequential validation process was attempting to identify in serum the corresponding proteins for our candidate genes and assess their potential use for early diagnosis. We also included a subgroup of patients with adenoma, since this is also an important target for CRC screening. Using commercial ELISA kits we could assess the protein levels of all the genes prioritized. Remarkably, COL10A1 showed relevant enough differences between controls and colon cancer patients (p = 3.2×10^−6^) to be proposed as a potential diagnostic candidate. MMP7 also showed some differences for adenomas (p = 0.0092), but showed an opposite direction to the expected one.

We have identified the protein COL10A1 which, when detected at high concentrations in blood, may be indicative of the presence of a neoplastic lesion in the colon. This protein was selected after a sequential procedure in which we started exploring whole genome expression data in colon tissue. Elevated serum levels of COL10A1 were observed both for adenoma and colon cancer patients. The area under the ROC curve was 0.76, which makes COL10A1 as a promising diagnostic biomarker. The cutpoint of 280 ng/ml attained 0.63 sensitivity and 0.85 specificity for colon cancer or adenoma (Figure 4). Similar values were obtained for cancer only. A few cancer-free subjects showed high levels of COL10A1 in serum, higher than the average for adenoma, indicating that other processes not related to colorectal lesions can increase COL10A1 levels.

*COL10A1* is a short chain collagen mainly expressed by chondrocytes during ossification. Defects in this protein have been related to Schmid-type metaphyseal chondrodysplasia [35]. COL10A1 is not expressed in normal colon epithelium, but is a direct transcriptional target of *RUNX2* [36], a transcription factor that is expressed in cancer cells, and has been related to multiple cancers. The elevated expression of *COL10A1* observed in tumors might be an indirect effect of higher-level regulatory alterations occurring in the tumor. In fact, we have observed a high correlation between RUNX2 and *COL10A1* expression in tumors (Pearson R = 0.5, results not shown). Our expression data also identifies high correlation between *COL10A1* and other genes: *SFRP4*, *INHBA*, *TNFSF4* that are involved in cytokine and Wnt signaling [37]–[40]. Recently *COL10A1* has been found to be overexpressed in diverse tumors related to the vasculature component [41]. However, the expression in other tissues other that cartilage is low, which contributes to the specificity observed in our study. Moreover, a recent study suggests that *COL10A1* expression may be regulated by non-steroidal anti-inflammatory drugs [42], which have protective effect on CRC risk, suggesting that the mechanism of *COL10A1* overexpression might be also related to inflammatory processes. Specifically, we have also explored if COL10A1 levels were related to non-steroidal anti-inflammatory drugs consumption, as indicative of inflammatory conditions, but we could not find a strong association (Table S1).

Since the sample size used for the serum assays in our study is limited, further validation studies are needed to confirm that *COL10A1* is useful for population screening. However, the large differences observed among colon cancer patients and cancer-free controls position this biomarker as a promising candidate. We have adjusted the analyses for age, gender and batch effect to control for potential confounding. This was only relevant for MMP3, which showed a strong association with gender. COL10A1 also shows an association with primary tumor (T) reinforcing the idea that the association could be an additional link with colon cancer and tumor size (Figure S5) more than stage, showing no association. Other potential confounders explored, including multiple epidemiological factors and tumor characteristics, were not associated with serum levels of the different markers (Table S1).

Our findings evidence that serum biomarkers for CRC screening can be identified and may change the scenario in a near future. Other blood molecular markers can also be of interest. Detection of DNA methylated septin 9 (*SEPT9*) gene is a promising candidate in the development of a non-invasive molecular screening method [43], [44]. Although the *SEPT9* assay successfully identified 68% of colon cancers at a specificity of 89% [45], the cost of the test is high since it involves DNA extraction and a quantitative DNA methylation assay. Besides, the method of assaying DNA methylation is still a handicap for the creation of a robust diagnostic tool, since a quantitative PCR step is often required. Another biomarker for colon cancer is the fecal detection of aberrant methylation of Vimentin gene (*VIM*). In this case the authors report a sensitivity of 46% for a specificity of 90% [46]. However, biomarkers based on serum proteins detected by conventional ELISA could be cheaper and more reliable to be used in a daily clinical practice and for population screening. The detection of the carcinoembryonic antigen [47] is one of the most widely used tumor markers worldwide, especially in CRC. Although in clinical use for almost 30 years, with clear value for prognosis and progression detection of CRC, the value of carcinoembryonic antigen in colorectal cancer screening is low mainly due to its low sensitivity (about 35%) and specificity (between 30 and 80%) [48]. Other candidate biomarkers have been proposed by other studies, such as metalloproteinases MMP7 [49] and MMP9 [50]. MMP7 was one of the potential biomarker that appears in our candidate list. In that study the authors reported a 58% of sensitivity and 100% of specificity, and the area under ROC curve was 0.81, but we have not been able to confirm these results with the commercial ELISA kit used. As the authors pointed, further studies are required involving larger numbers of subjects, to confirm these results.

Although many other biomarkers for colon cancer have been previously proposed [51]–[54], in most cases there is no further progress beyond the proposal [2], [6], [11], [55]–[57] since none of them may have sufficient sensitivity and specificity to be considered in the current guidelines [13].

It is possible that other genes from the list of candidates identified in the expression analysis also could be useful for early diagnosis, either alone or in combination with *COL10A1*. Therefore, further studies are required to assess the utility of other potential biomarkers for the early detection of colorectal cancer.

In conclusion, after different steps of sequential validation, we have identified a list of candidate biomarkers for early detection of colon cancer. The most promising one is the detection of COL10A1 in serum, which can identify adenoma and invasive cancer with high sensitivity and specificity. The use of a cheap serum test for CRC screening should improve participation and contribute to decrease the burden of this disease.

## Supporting Information

Figure S1 **Strip charts of expression values for housekeeping genes in discovery series and technical validation series.**
(PDF)

Figure S2 **Density plots of expression values for housekeeping genes in discovery series and technical validation series.**
(PDF)

Figure S3 **Expression levels for the discovery series, technical validation and biological validation of the 23 selected genes.**
(PDF)

Figure S4 **Serum concentration values of COL10A1 in relation to tumor stage.**
(PDF)

Figure S5 **Serum concentration values of COL10A1 in relation to tumor size.**
(PDF)

Table S1 **Association of serum markers with patient characteristics, epidemiological factors and tumor characteristics.**
(DOCX)

Table S2 **Excel file with the complete list of candidate biomarkers identified in the discovery phase.** Sheet “T-F”: expression in tumor > expression in cancer-free mucosa; Sheet “A-F”: expression in adjacent normal mucosa > expression in cancer-free mucosa.
(XLS)
