# Supplementary figures and images for: Discovery and Validation of New Potential Biomarkers for Early Detection of Colon Cancer
Source: PLoS One. 2014 Sep 12;9(9):e106748. doi: 10.1371/journal.pone.0106748 (PMC4162553; doi:10.1371/journal.pone.0106748)

## Housekeeping genes – Discovery series

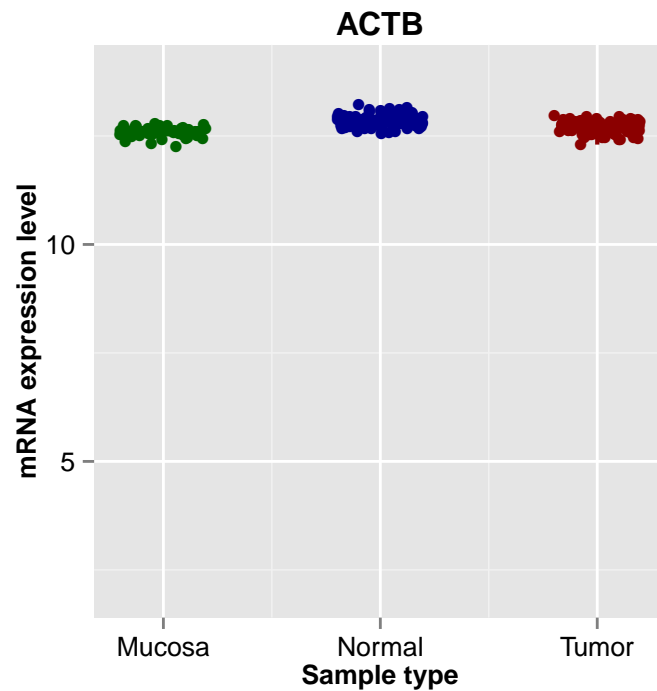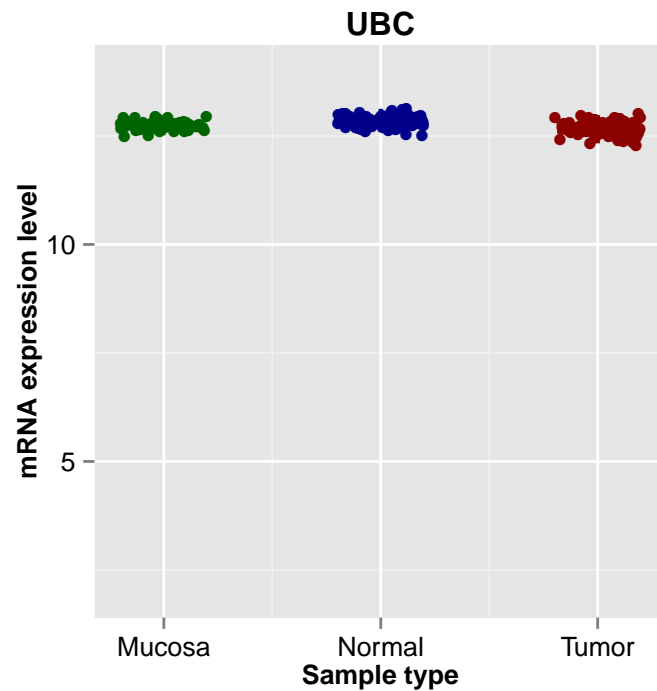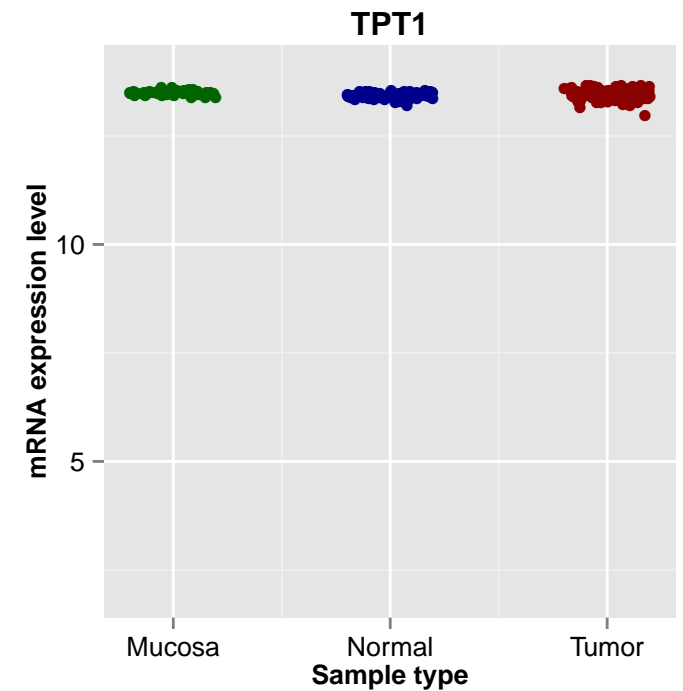

## Housekeeping genes – Technical validation

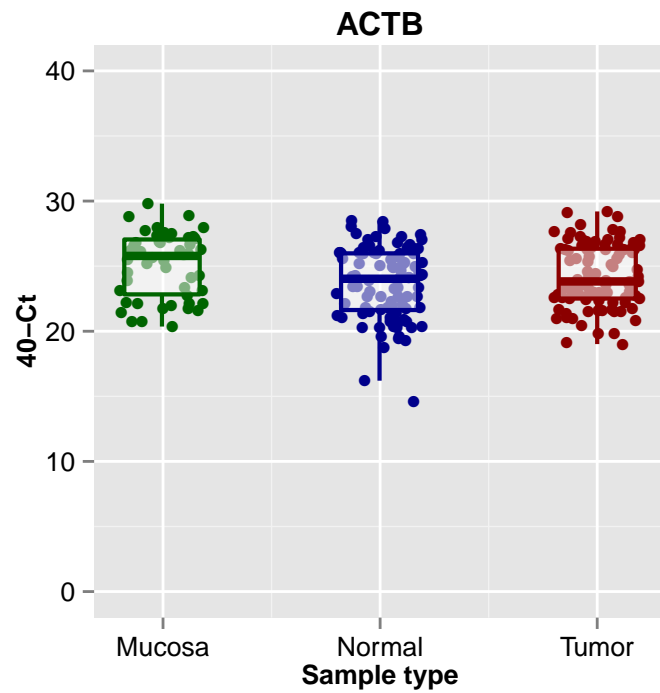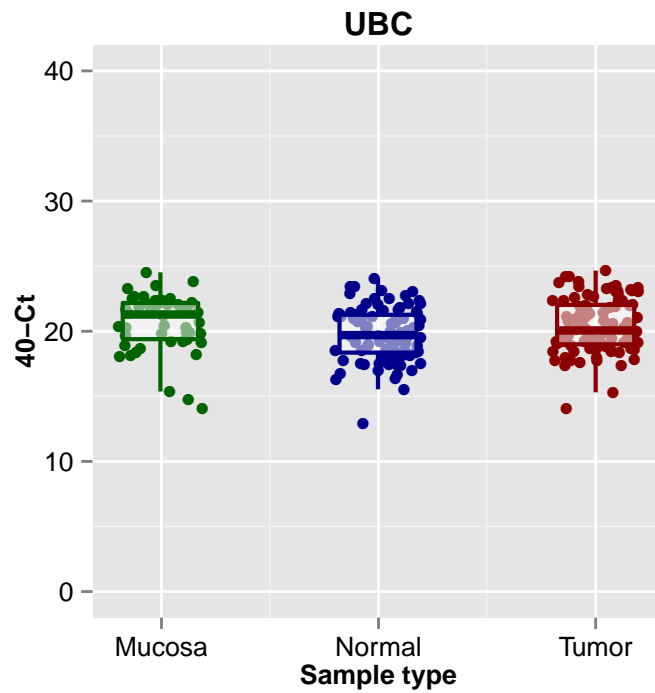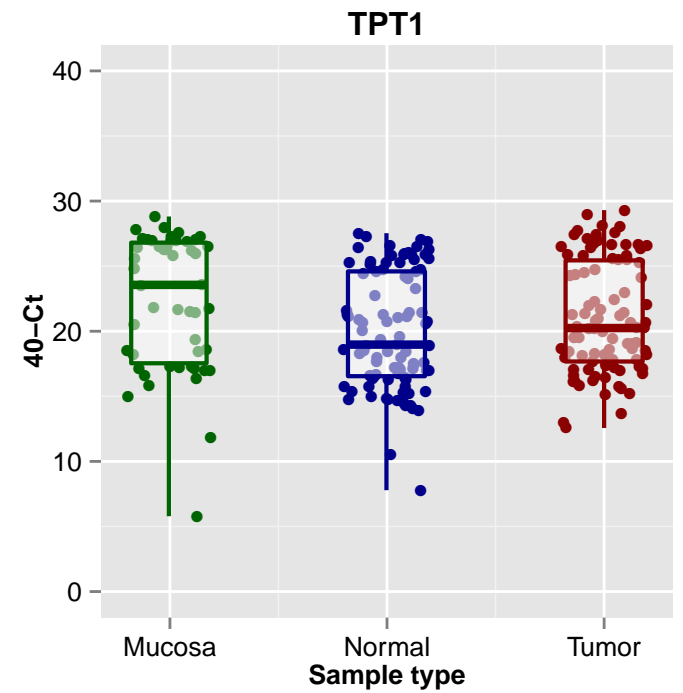

Supplement: Figure S1 — Strip charts of expression values for housekeeping genes in discovery series and technical validation series. (PDF) [file pone.0106748.s001.pdf]

## Housekeeping genes

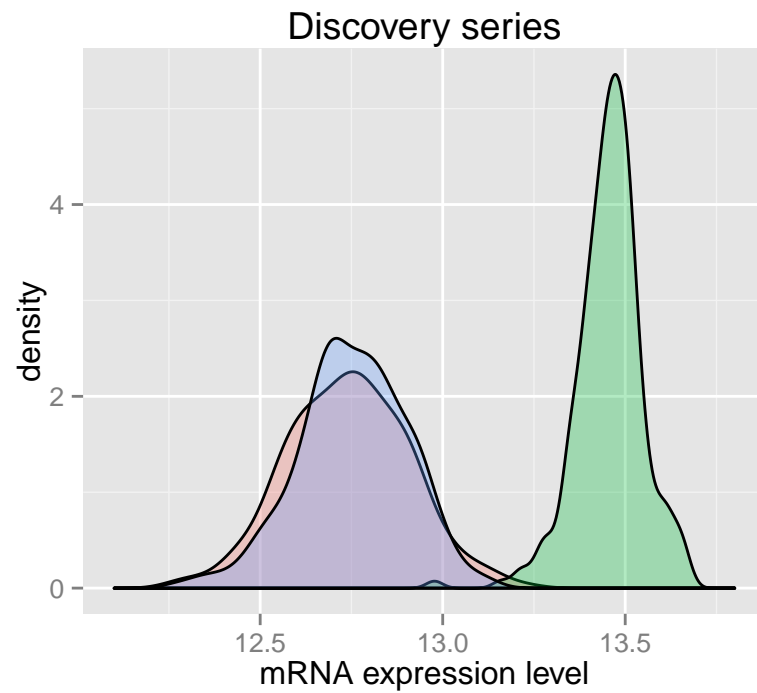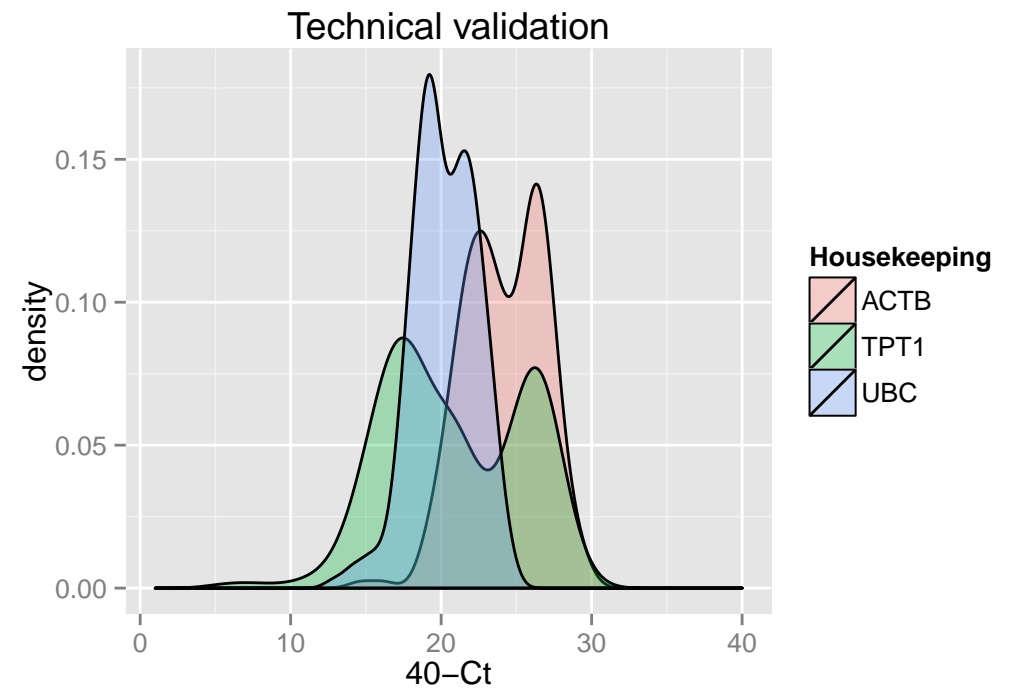

Supplement: Figure S2 — Density plots of expression values for housekeeping genes in discovery series and technical validation series. (PDF) [file pone.0106748.s002.pdf]

## CA9

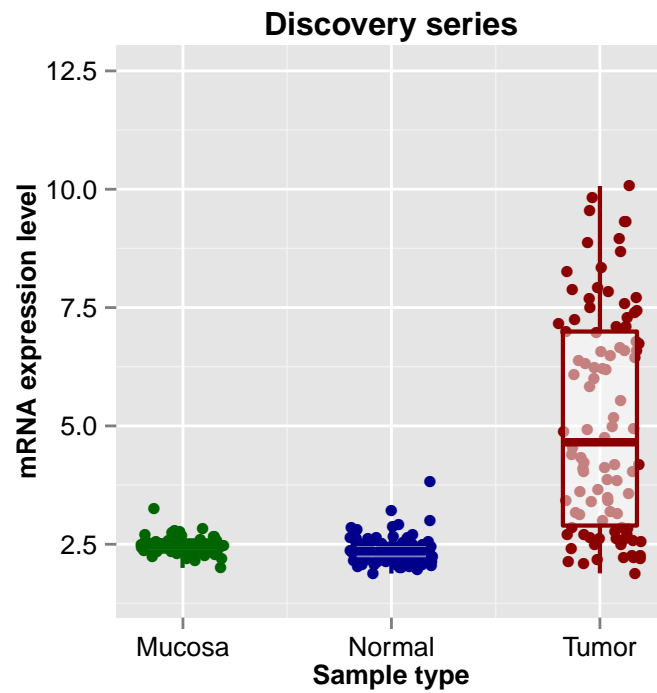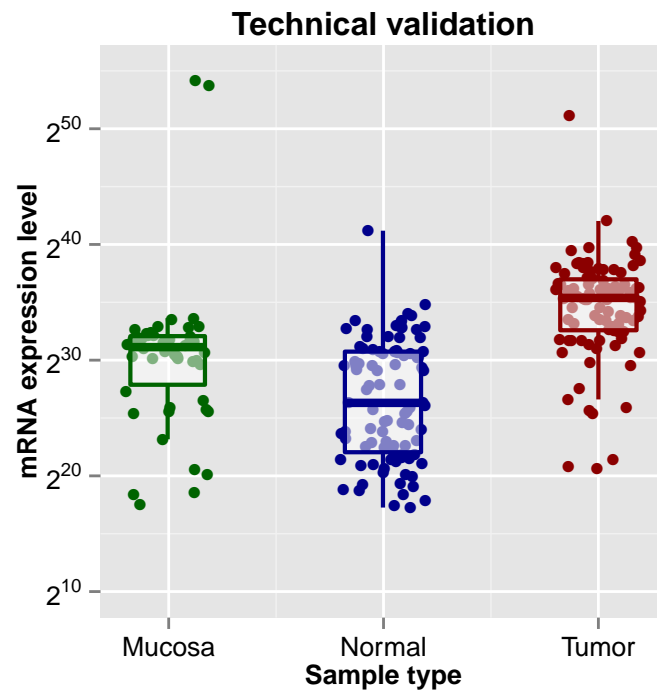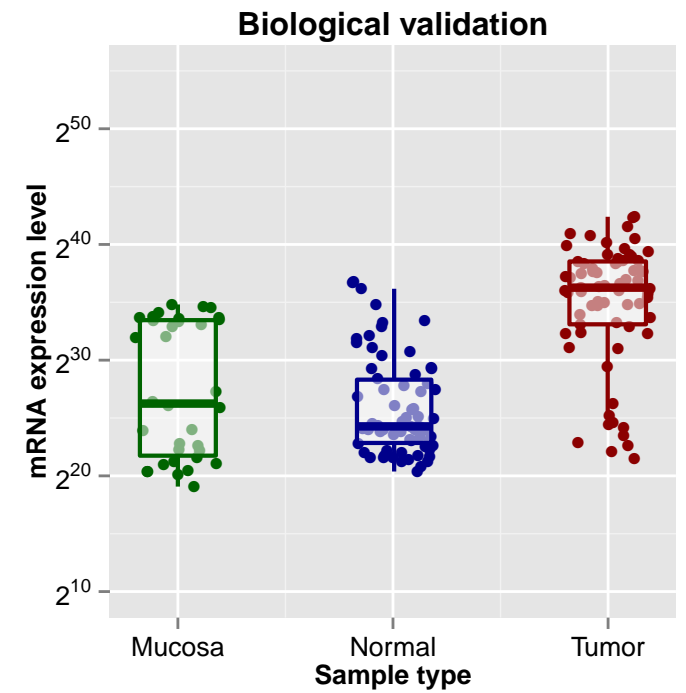

# CEL

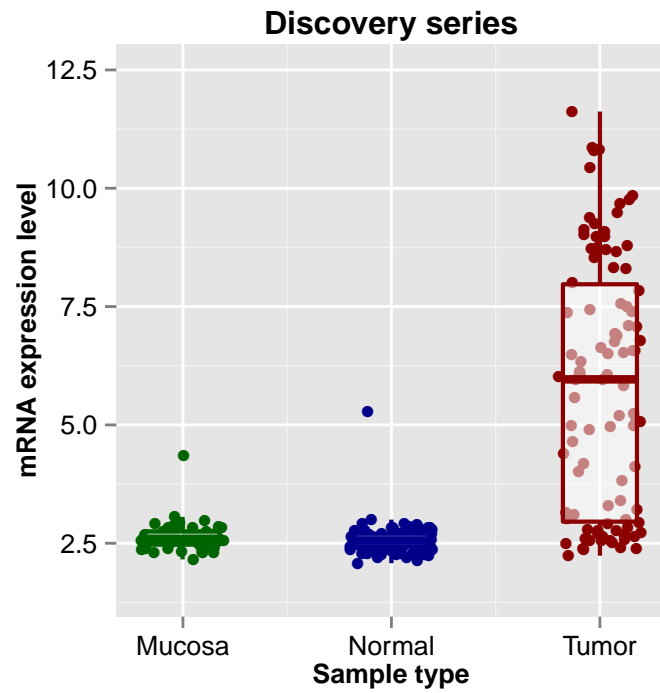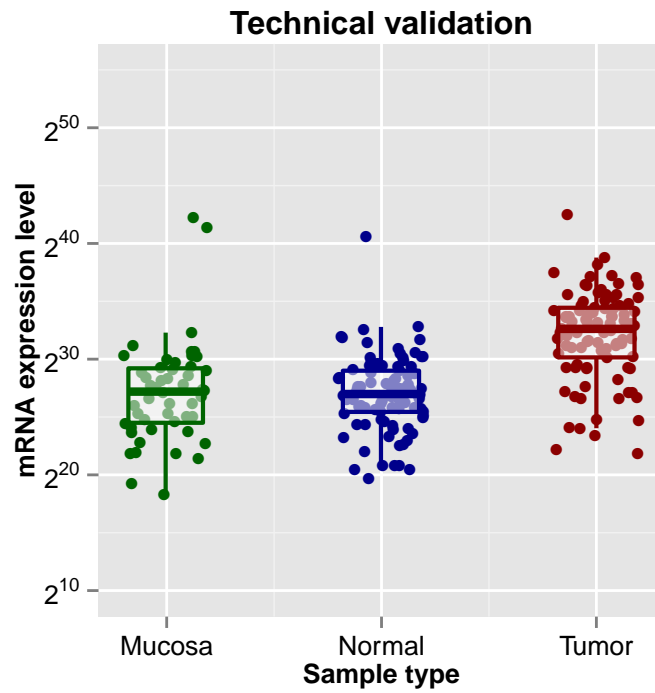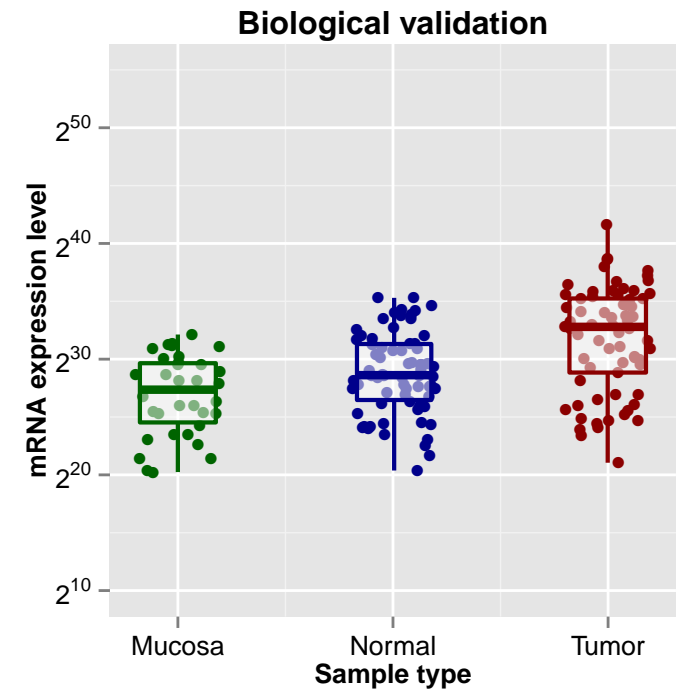

# CLDN2

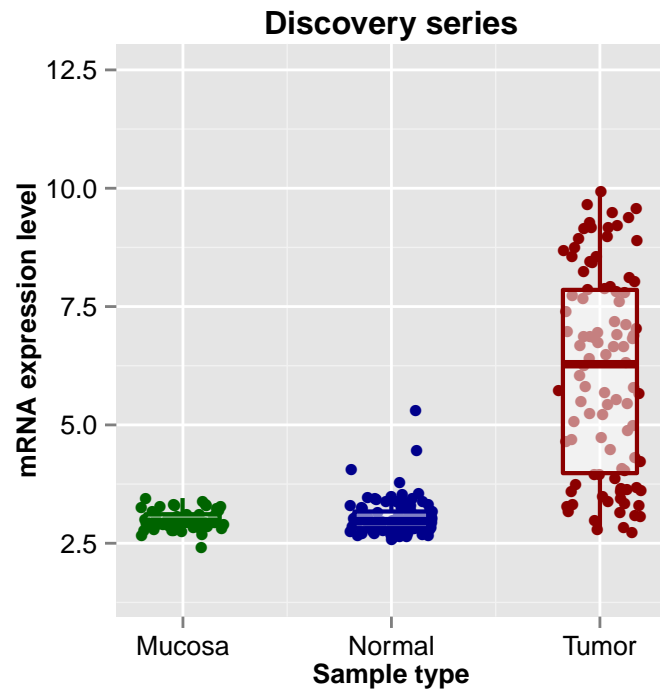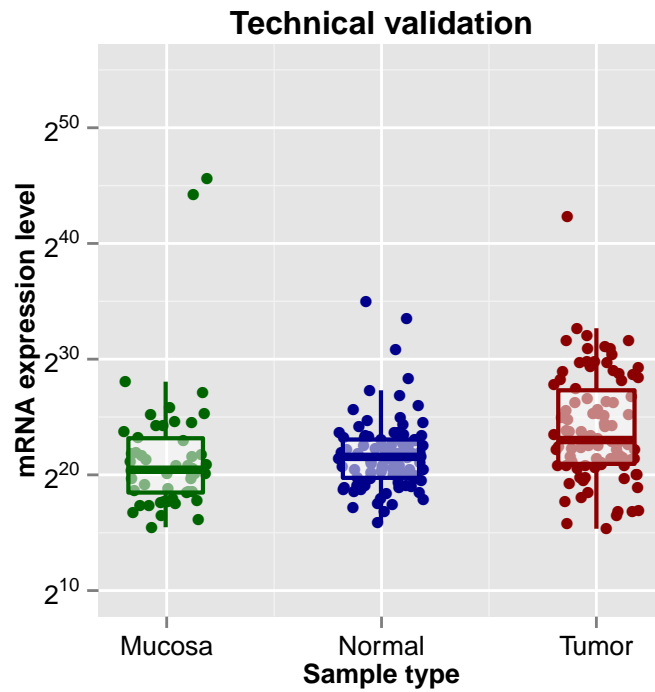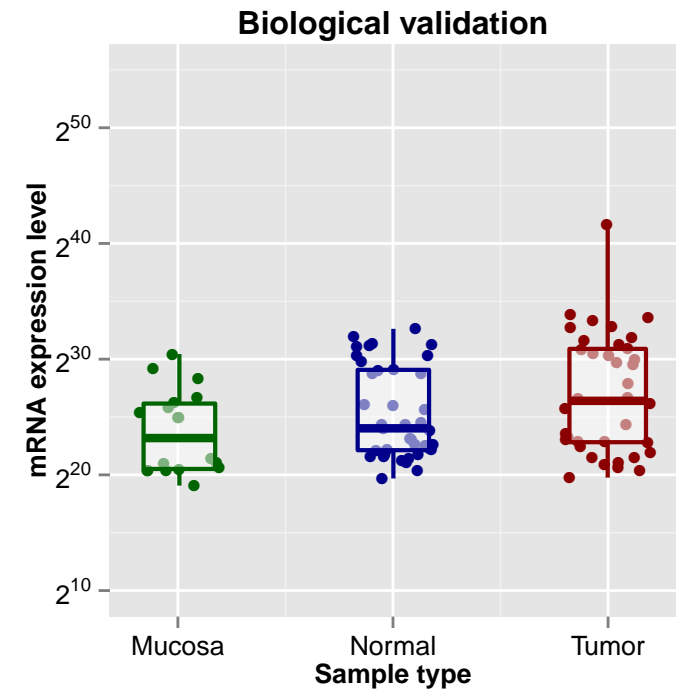

# COL10A1

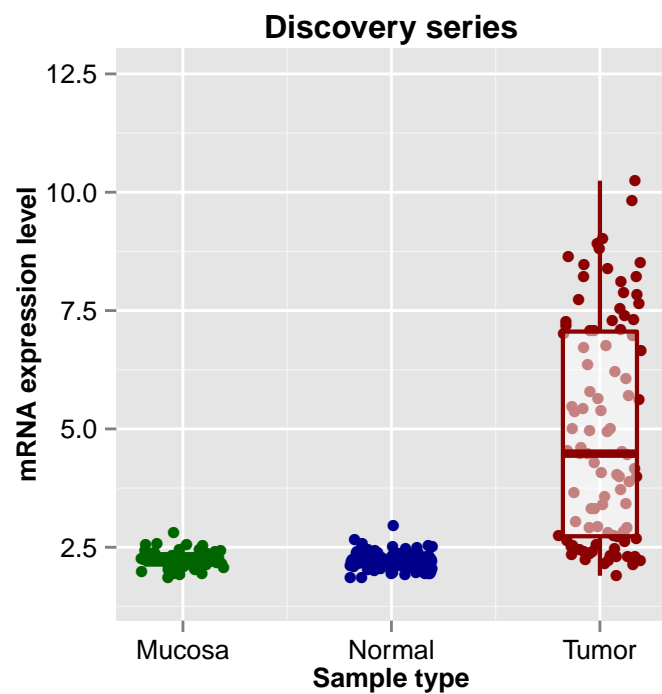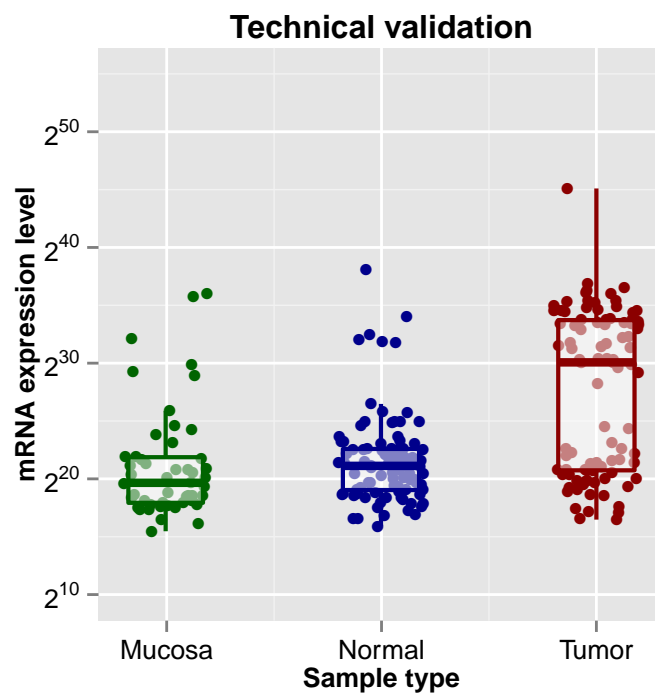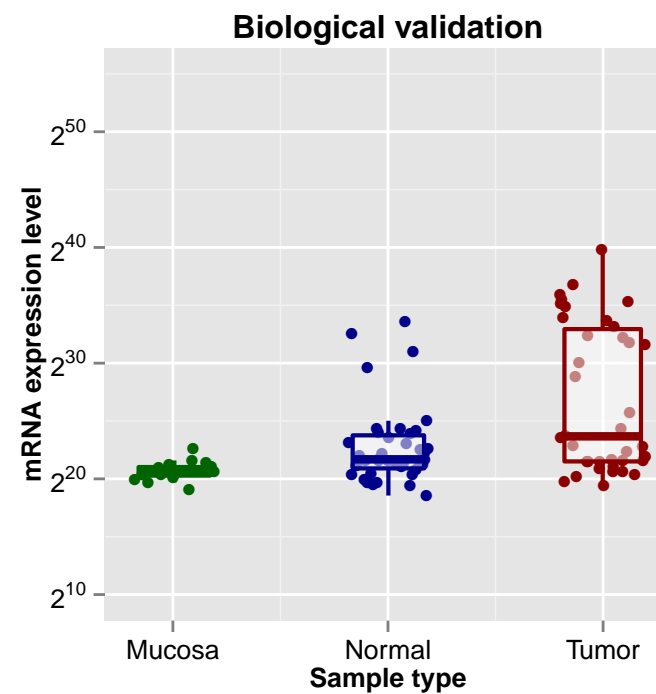

# COL11A1

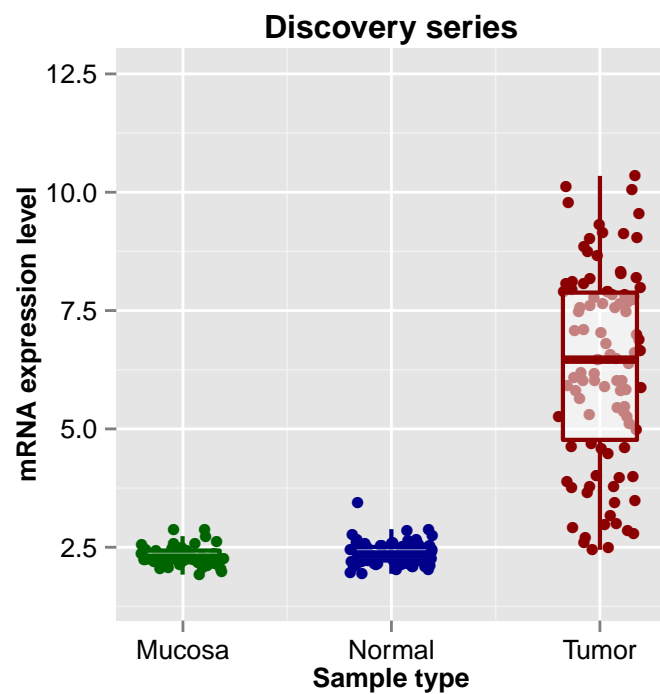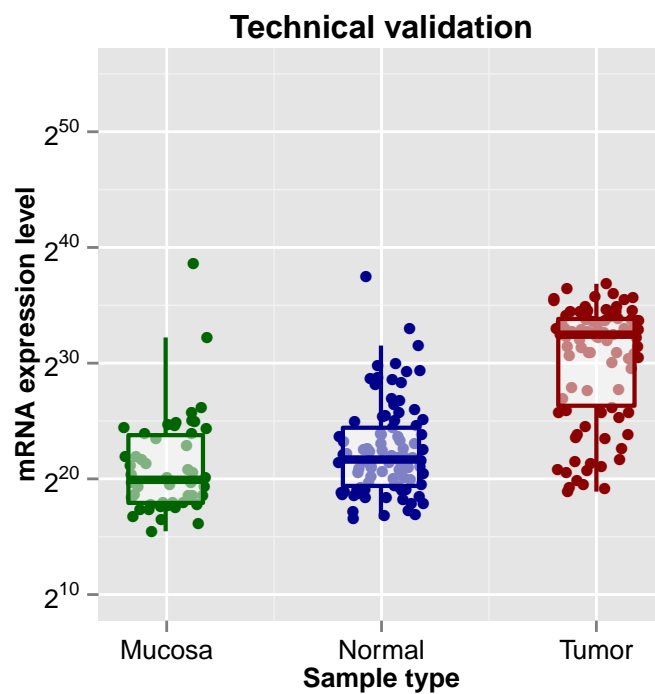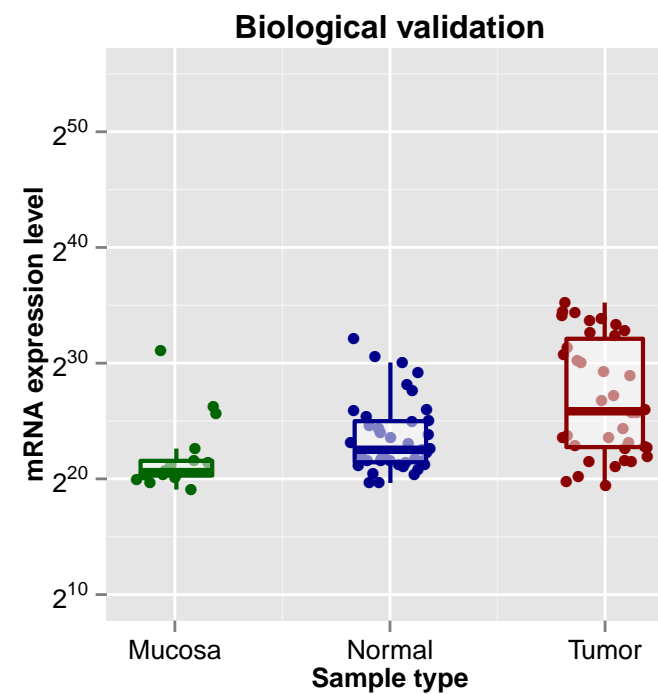

# CST1

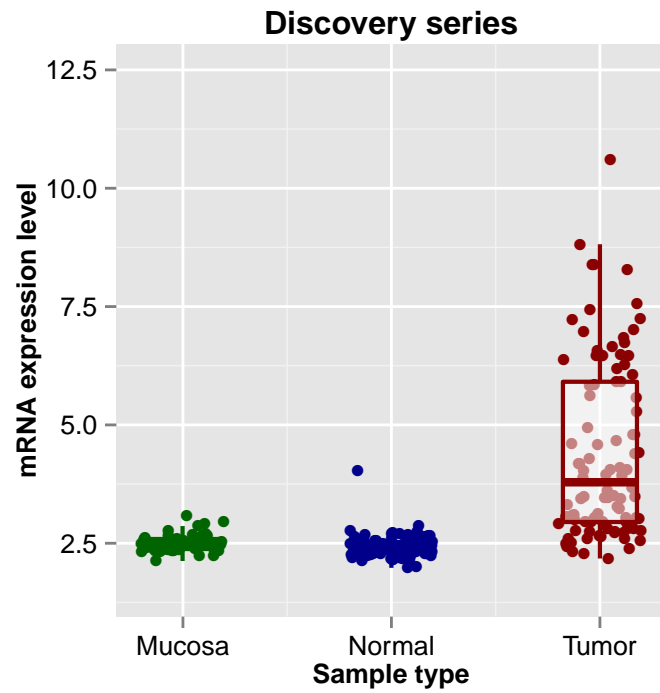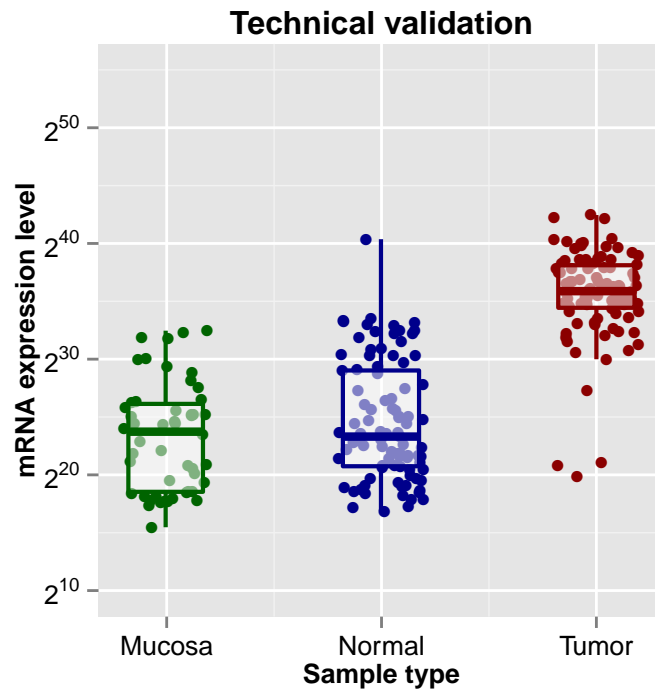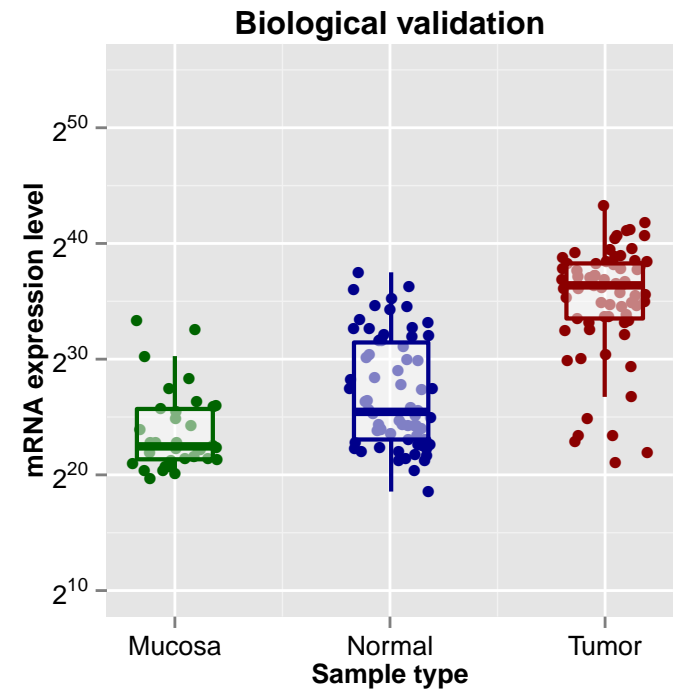

# CXCL5

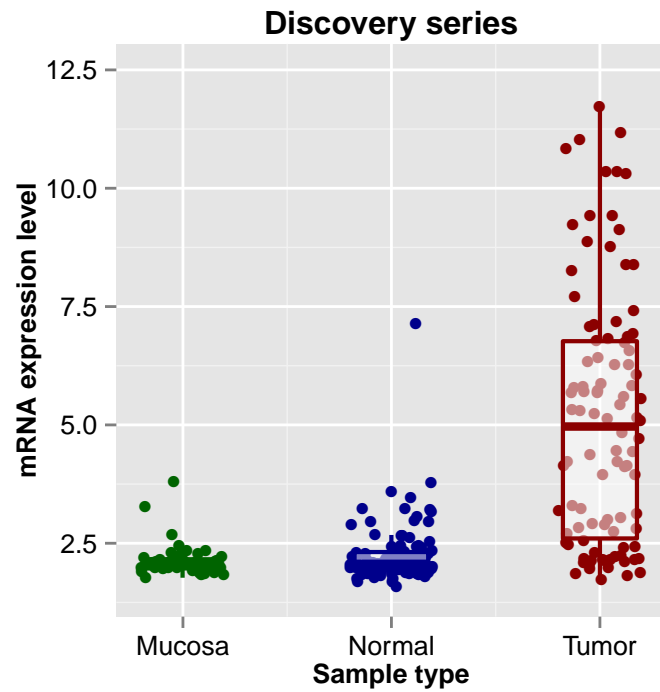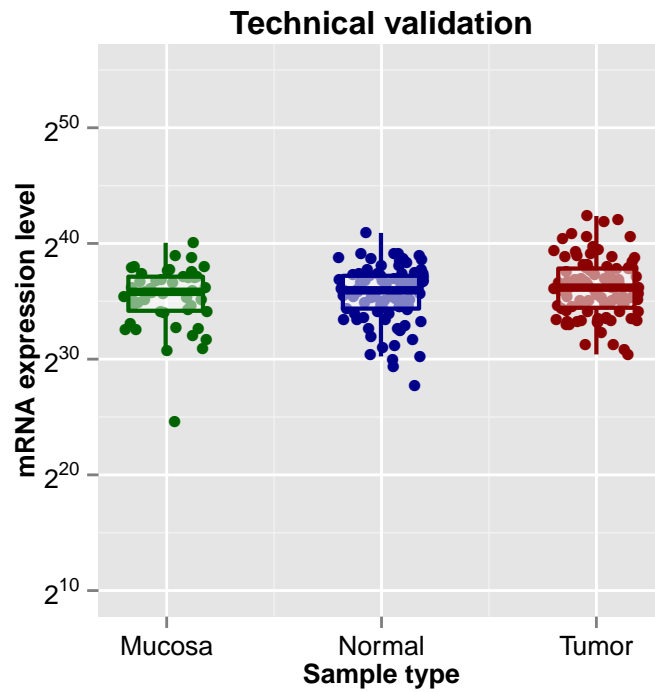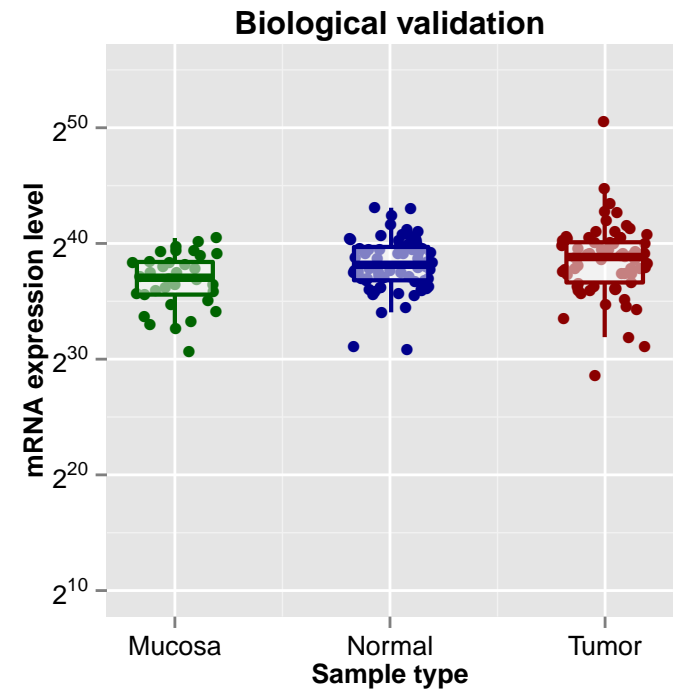

# DPT

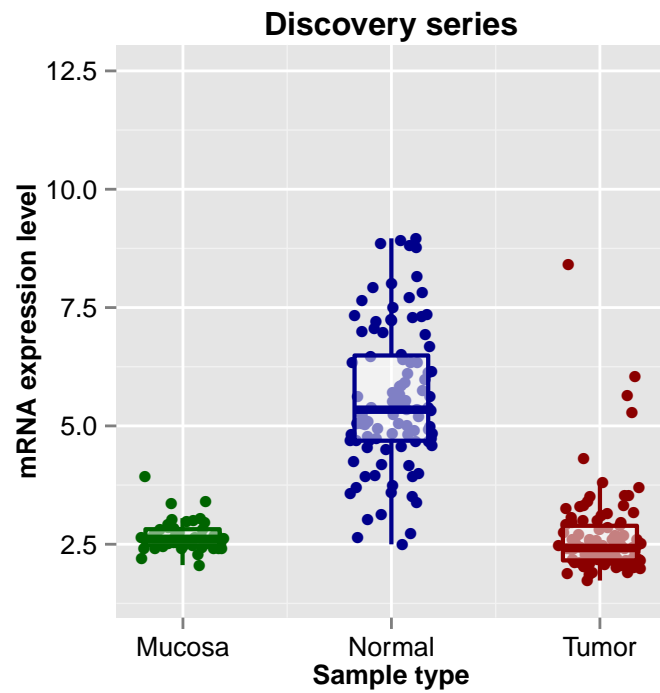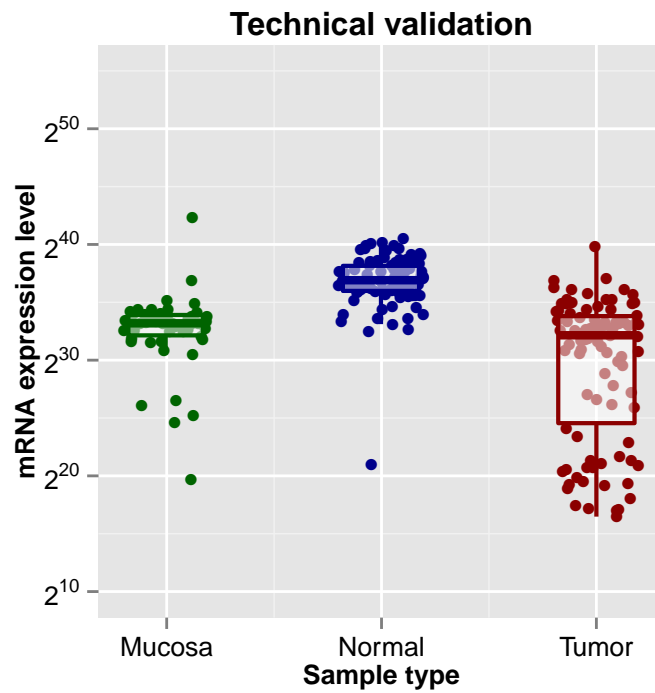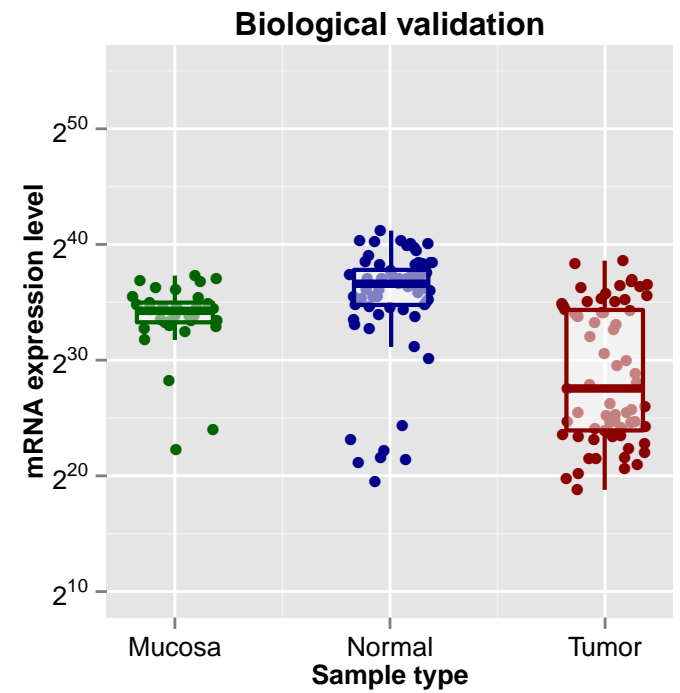

# EPHX4

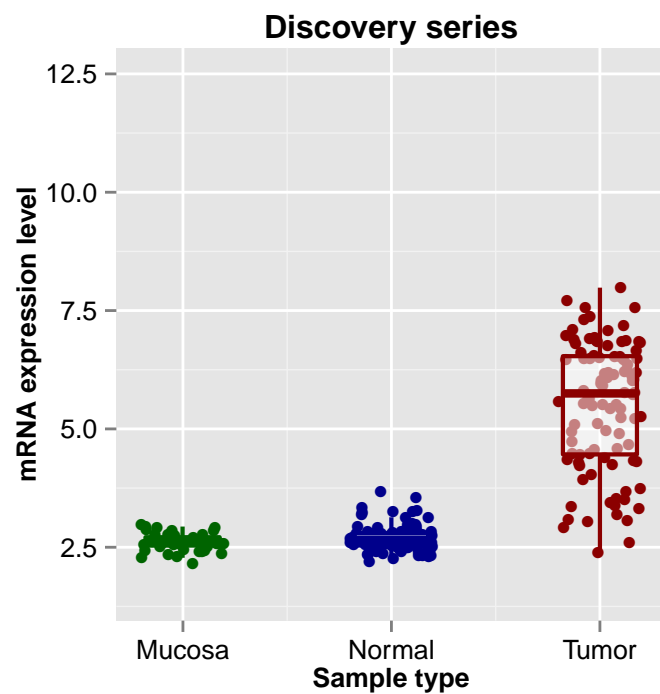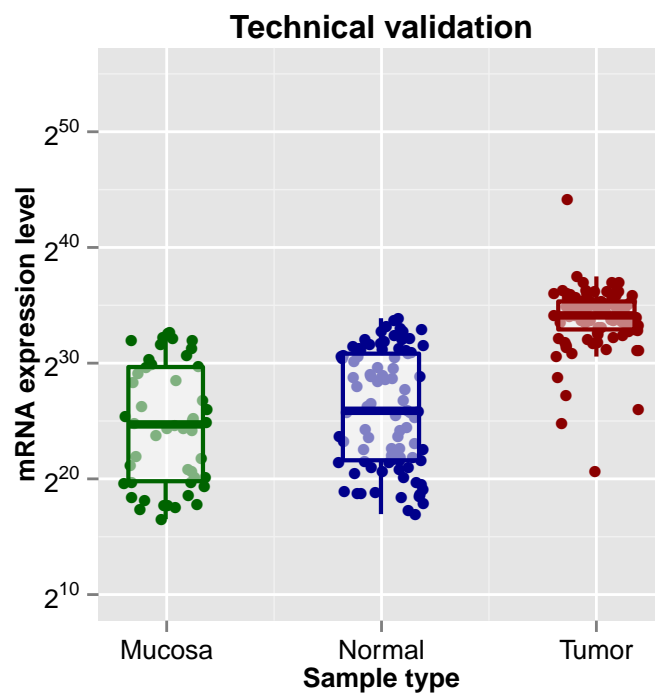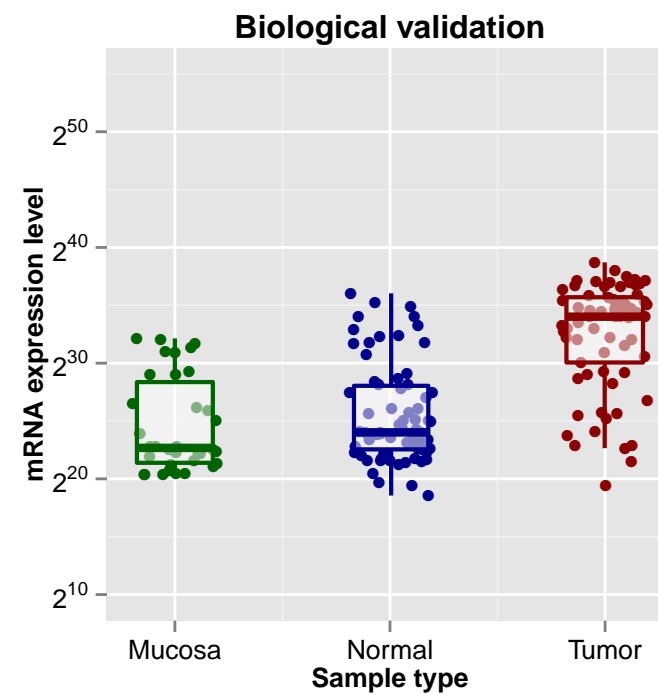

# ESM1

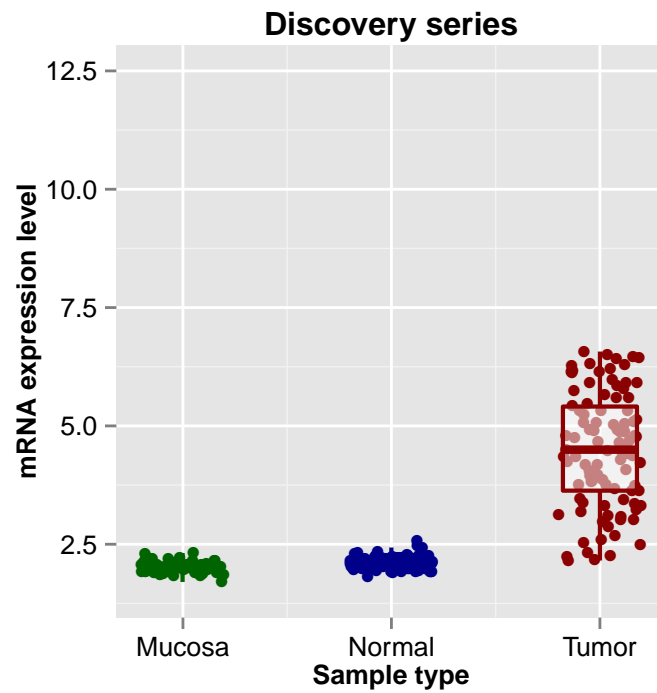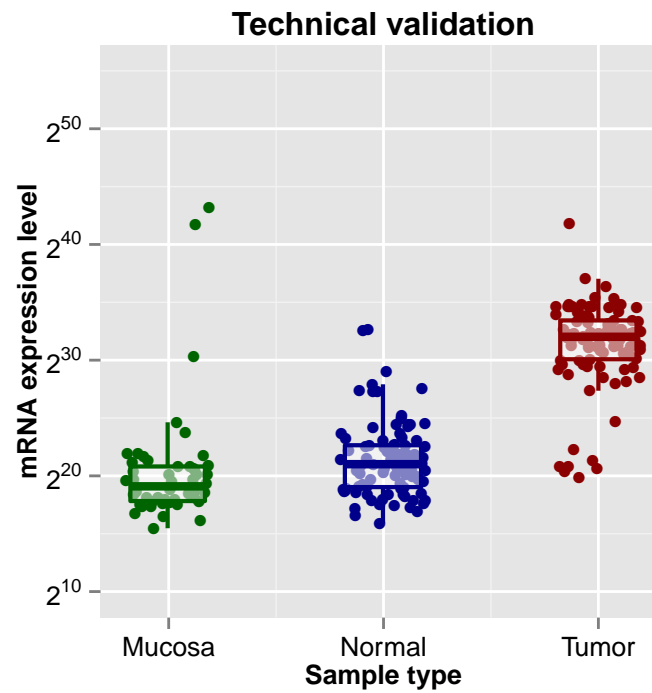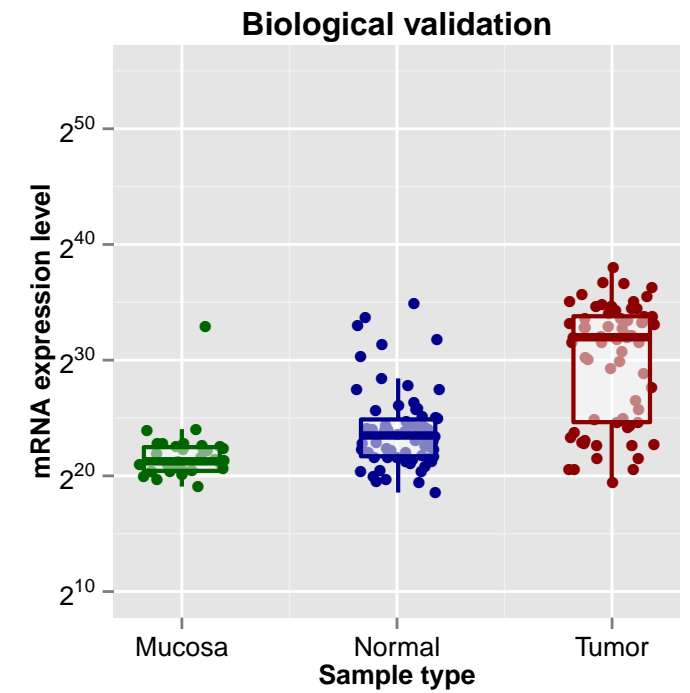

# FAP

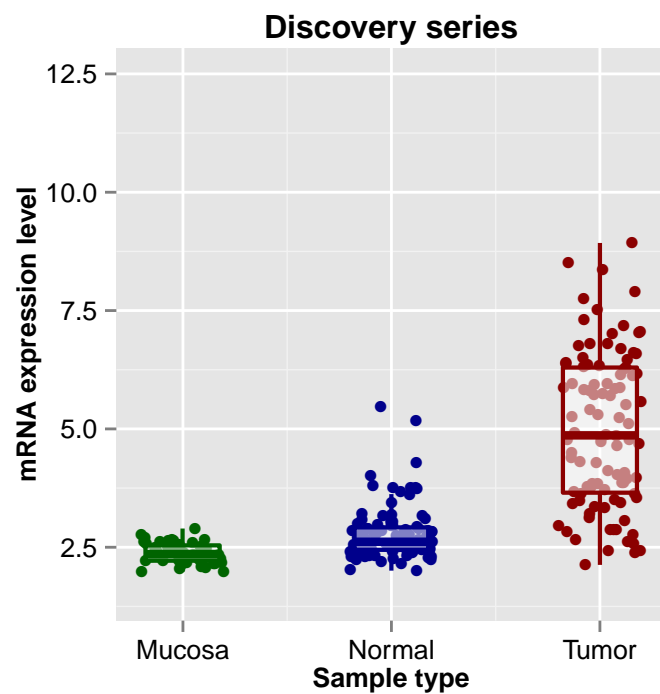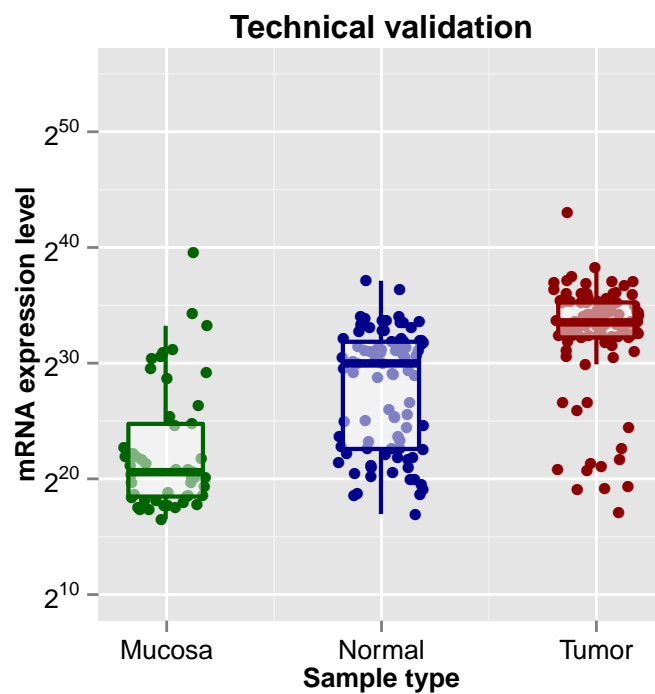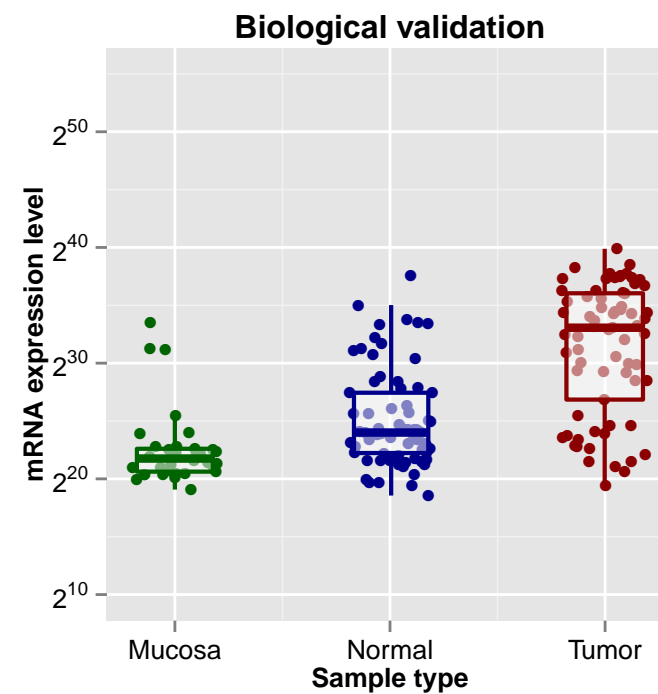

# GAL

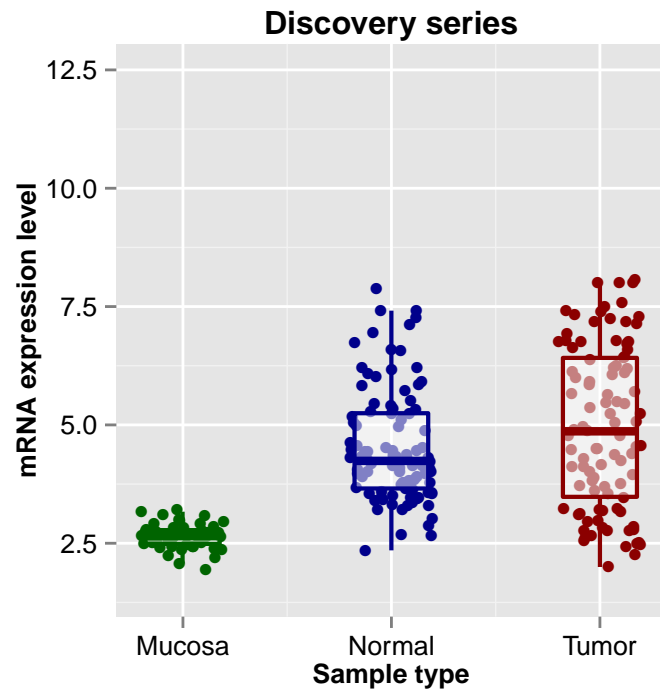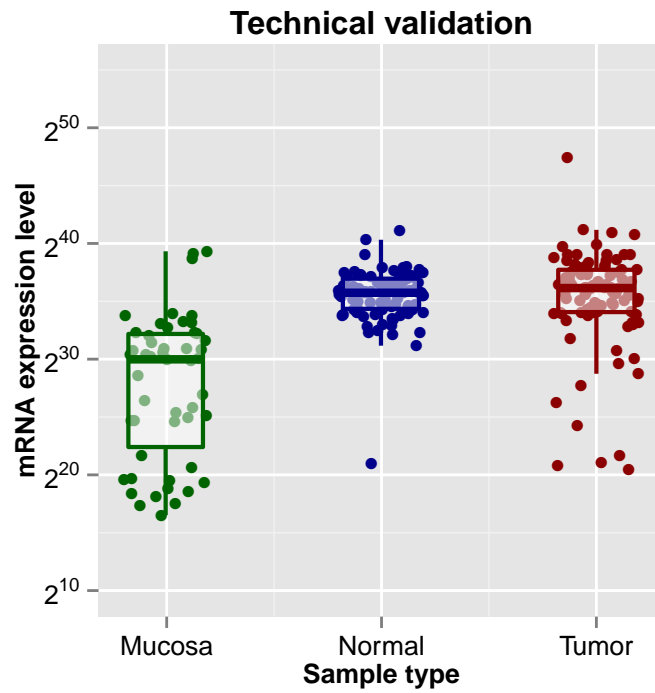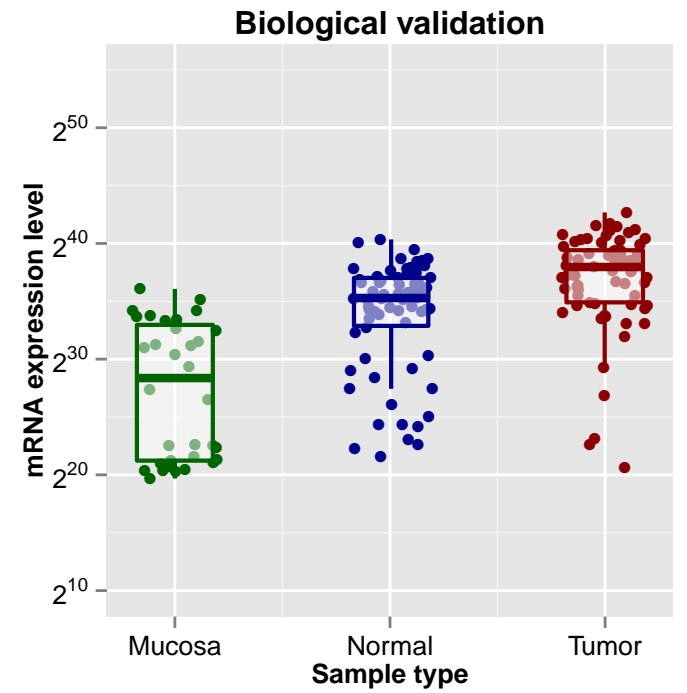

# JUB

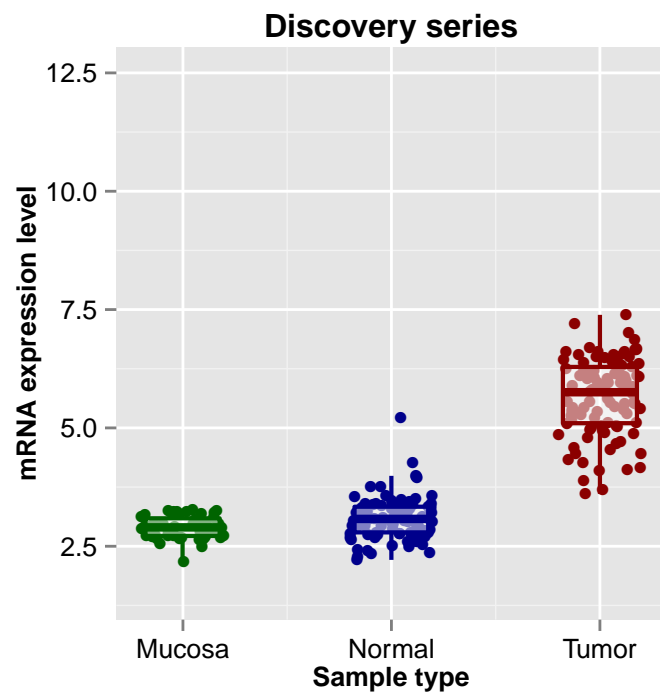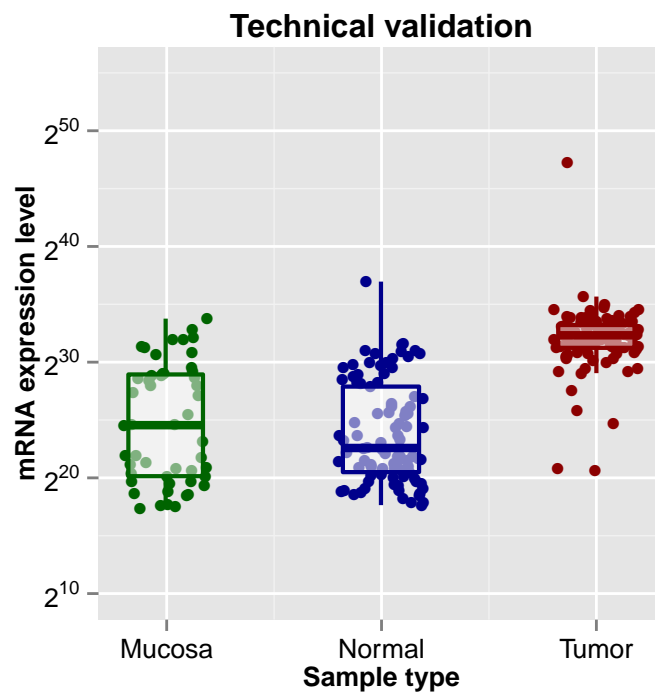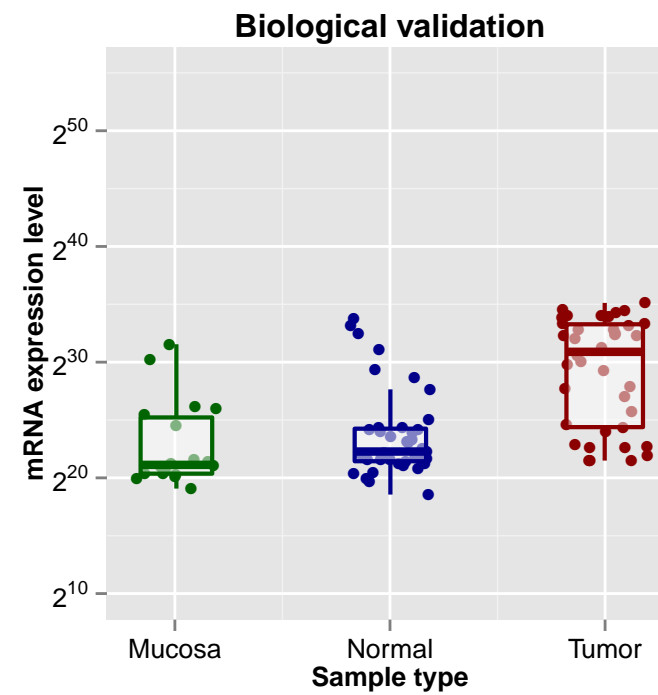

# KIAA1199

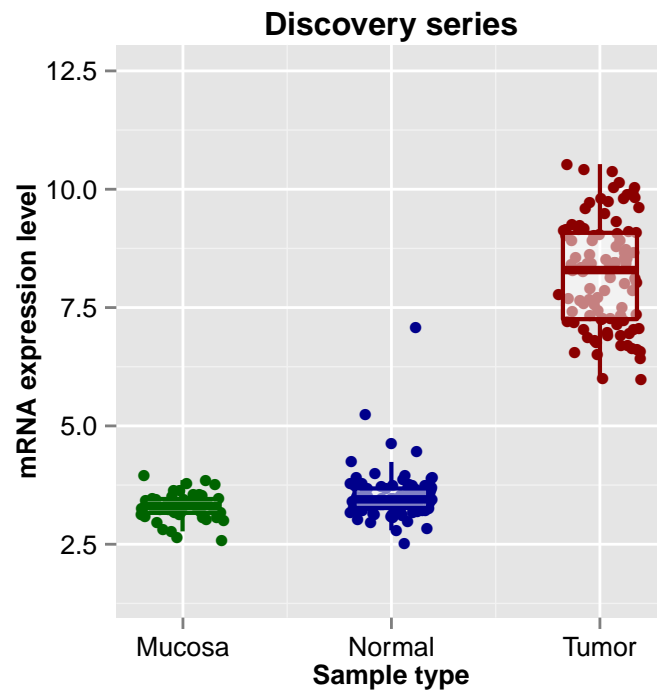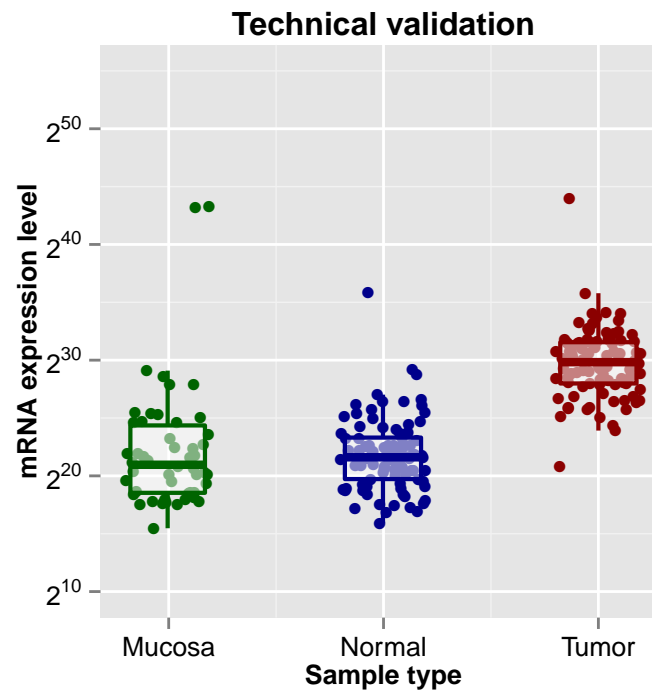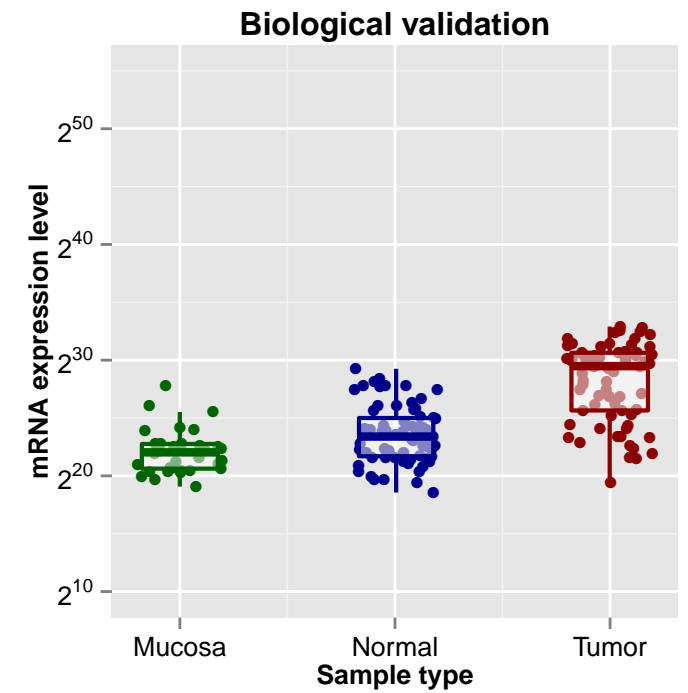

# LOC100127888

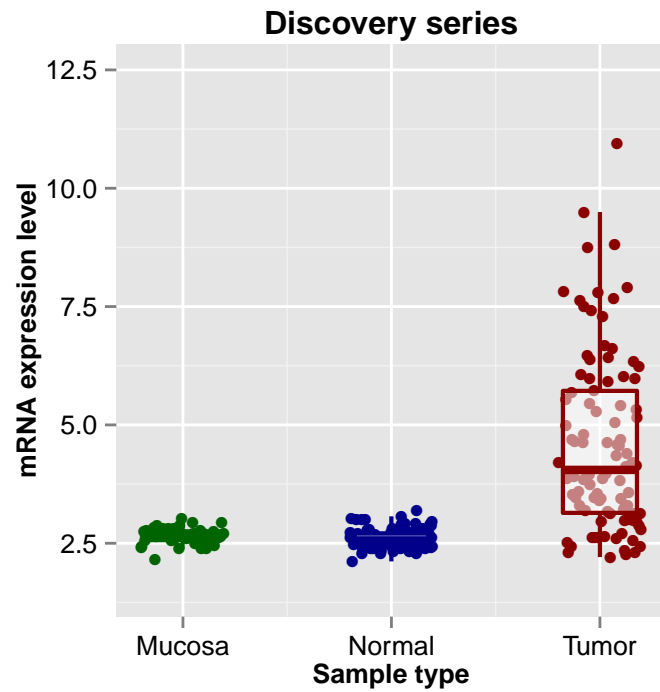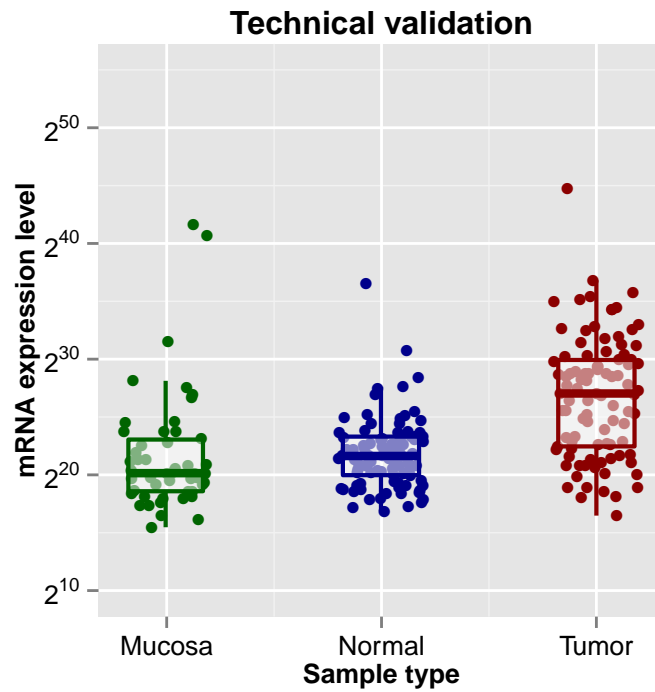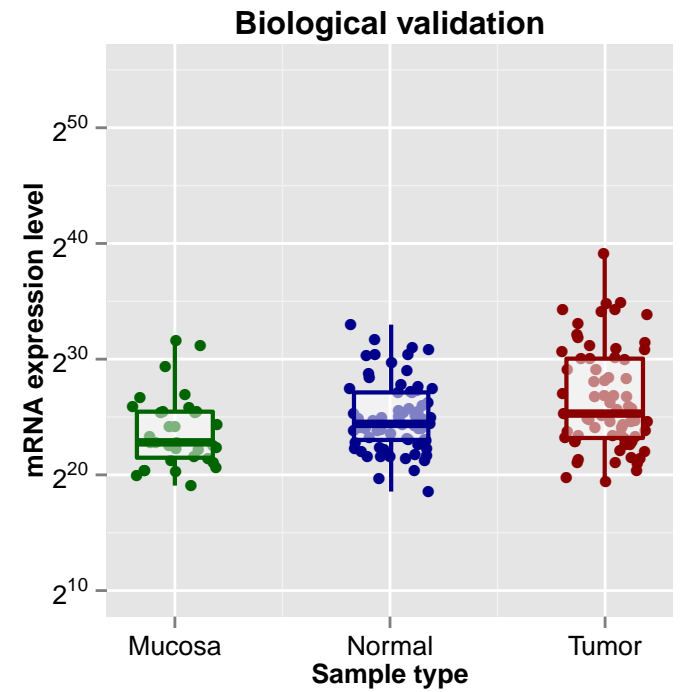

# MMP10

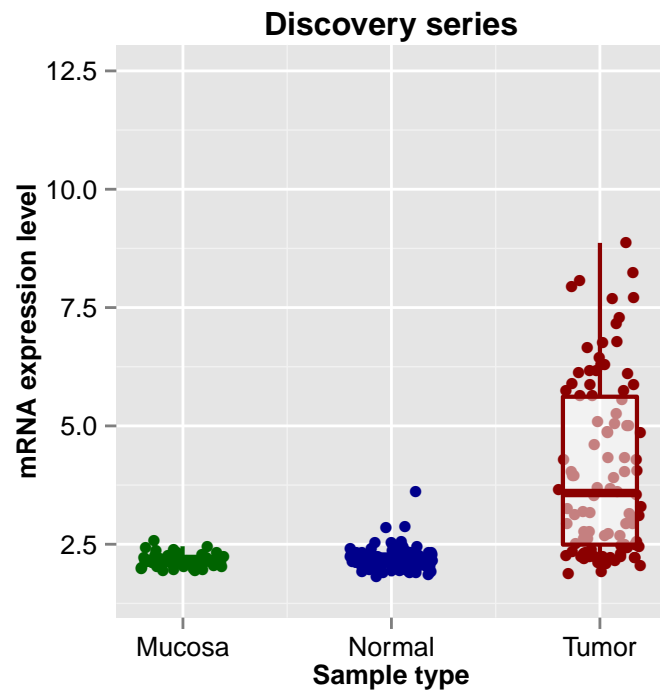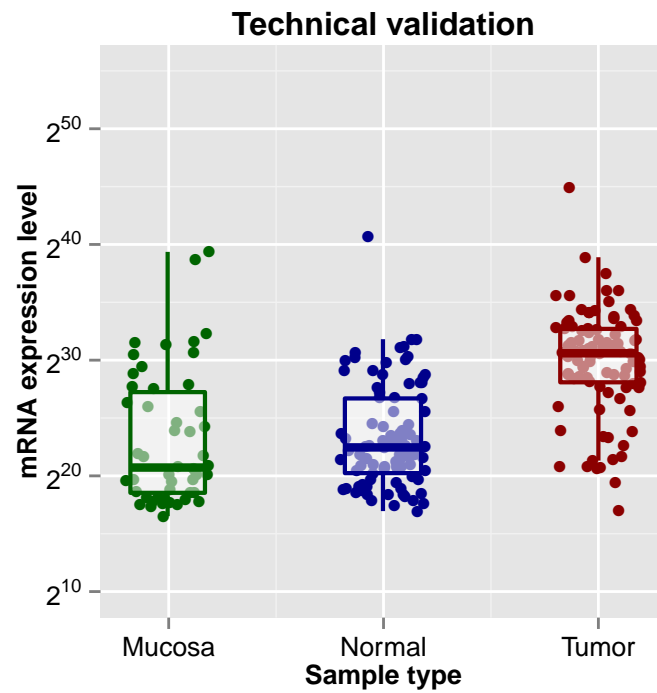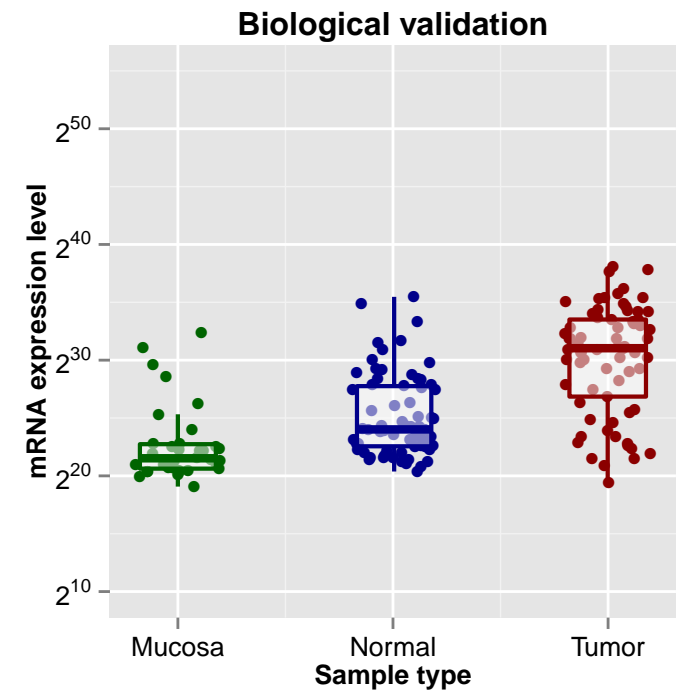

# MMP3

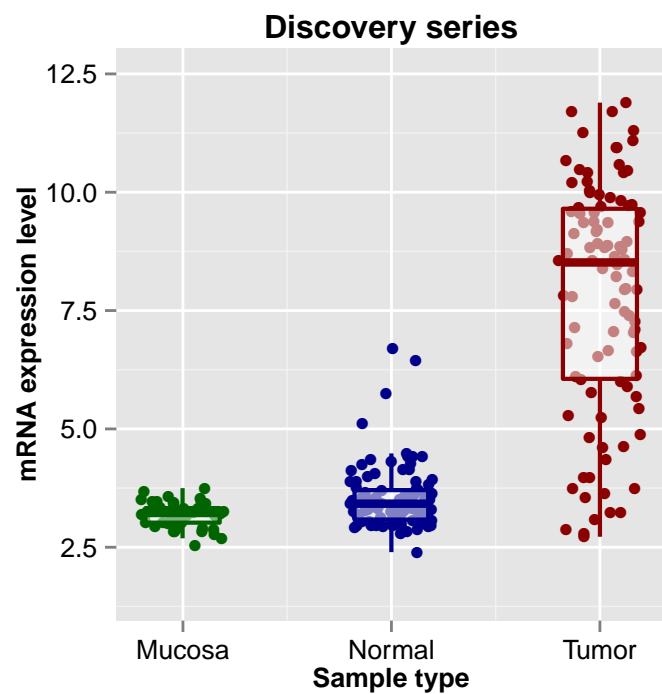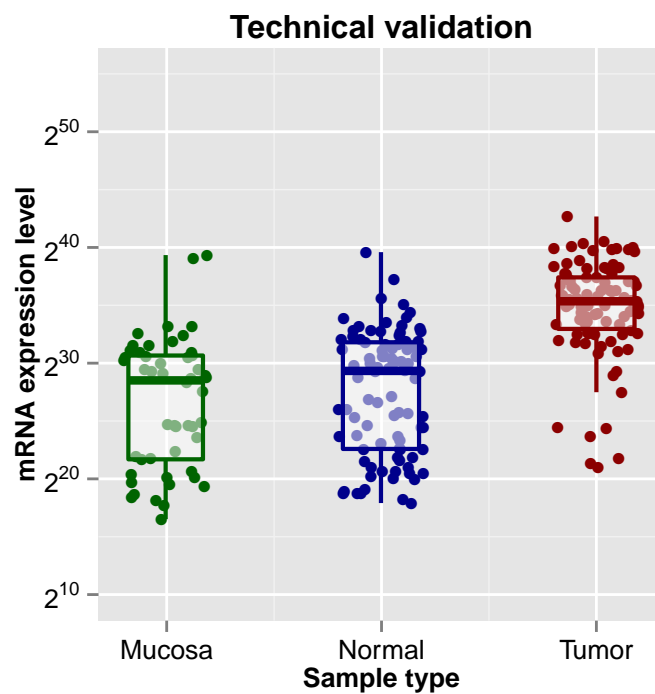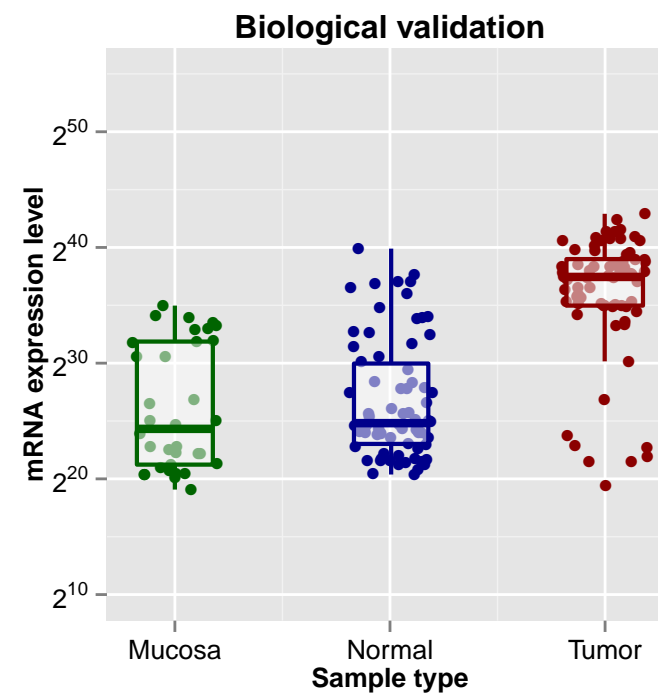

# MMP7

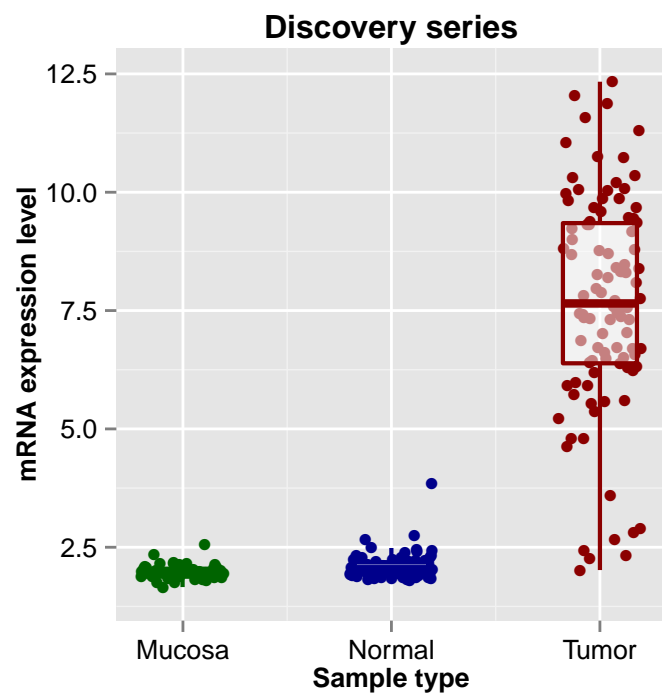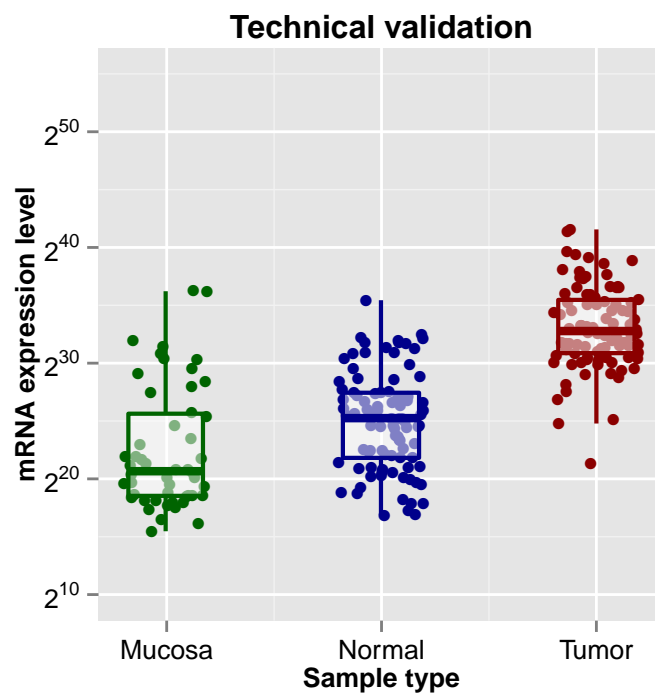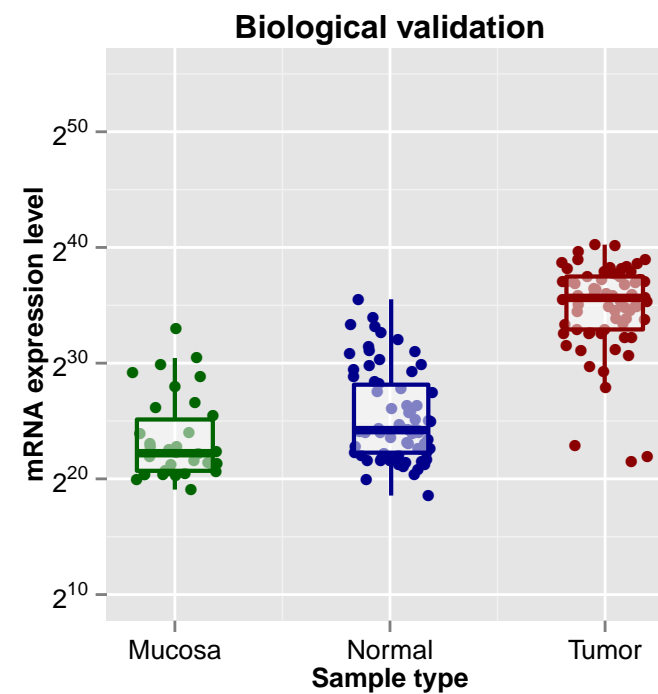

# MSX2

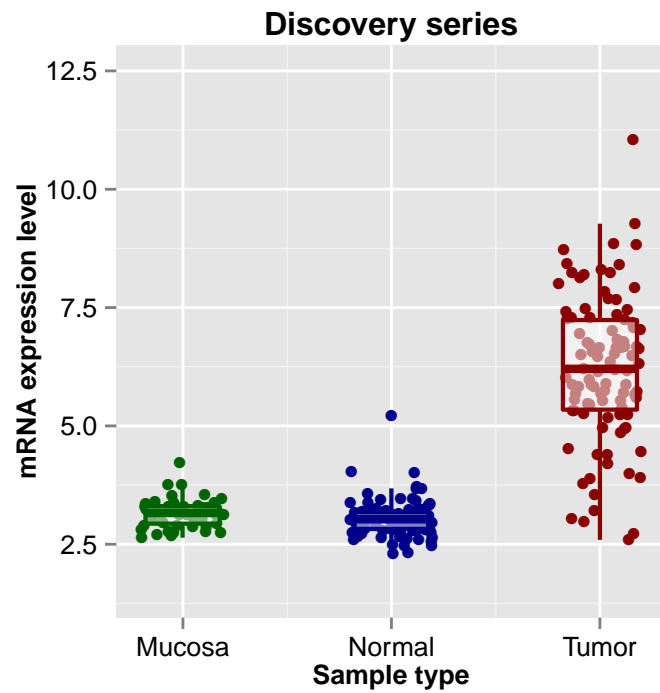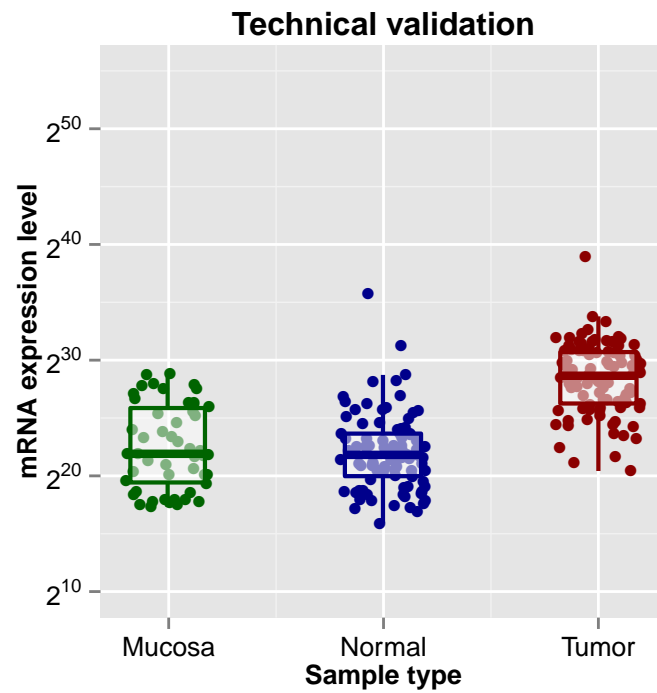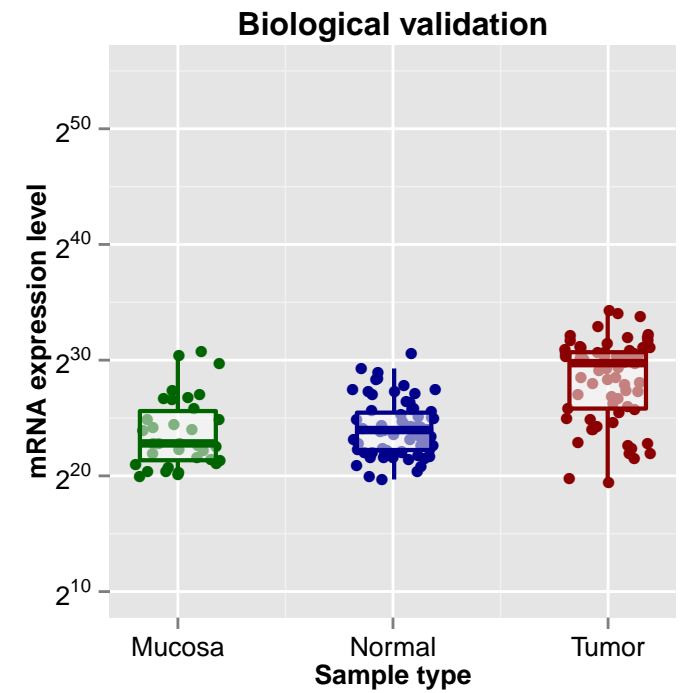

# SFRP2

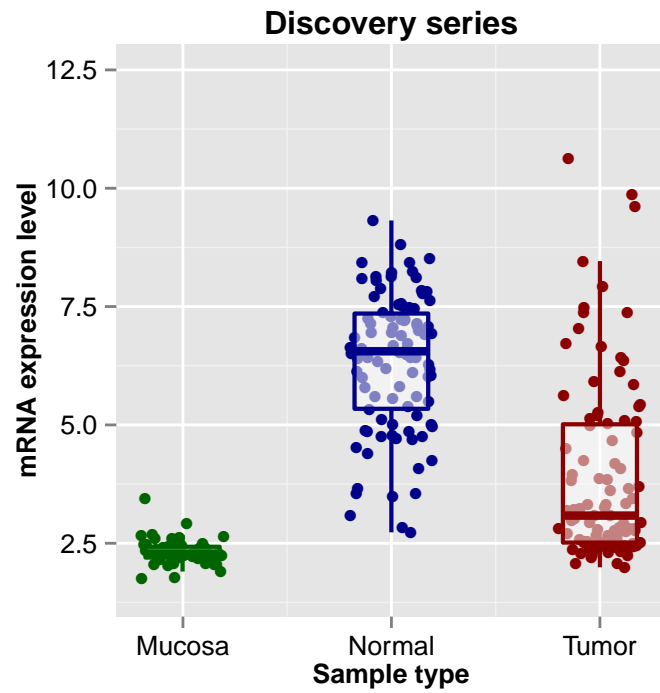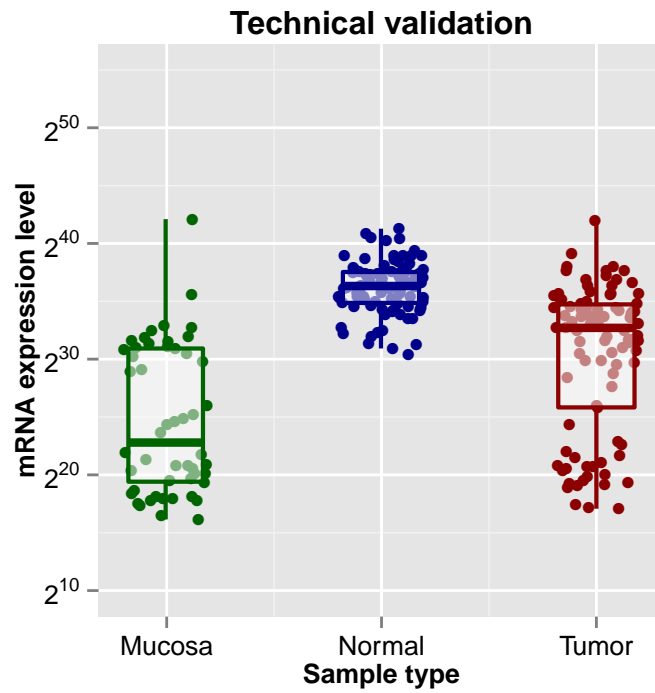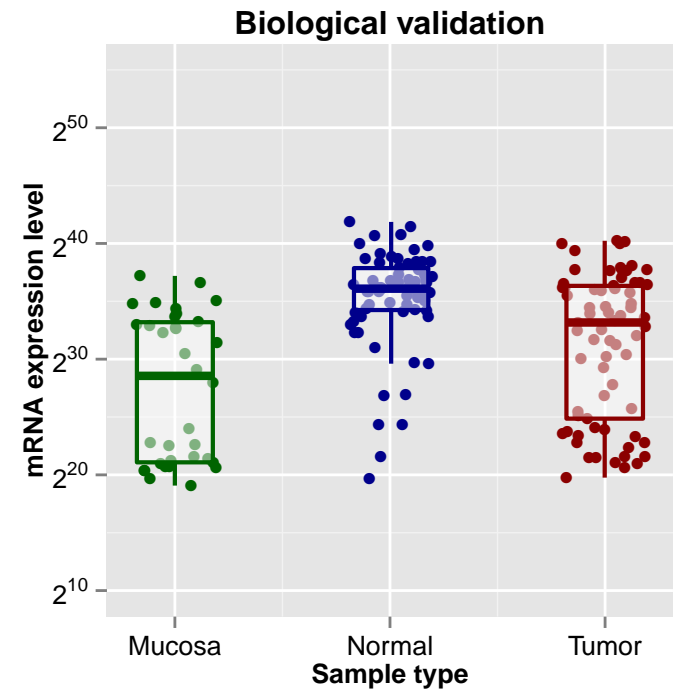

# SRPX2

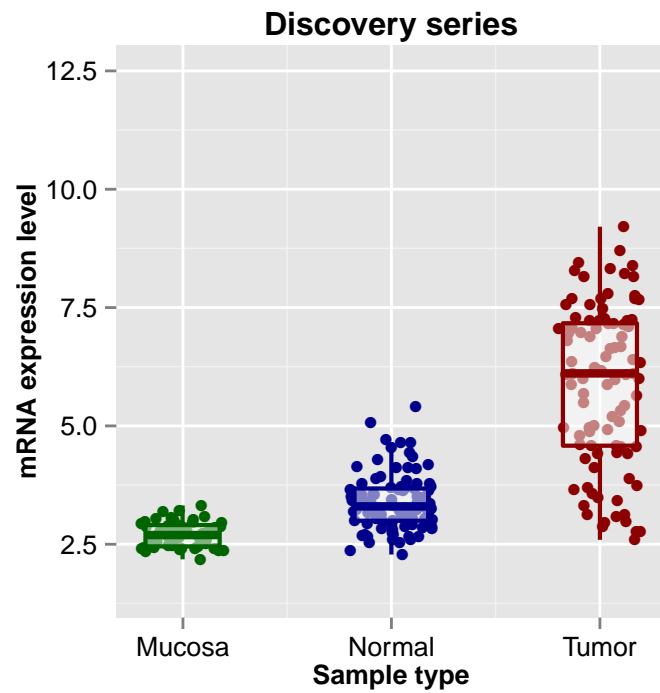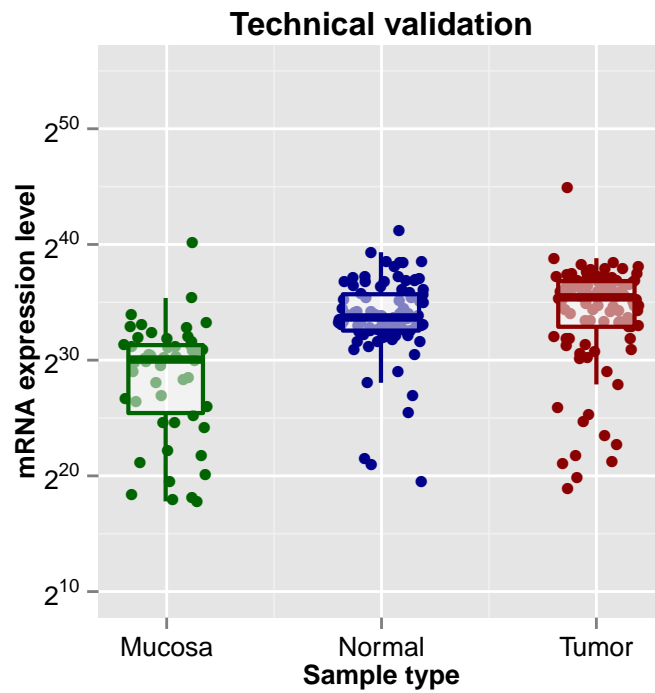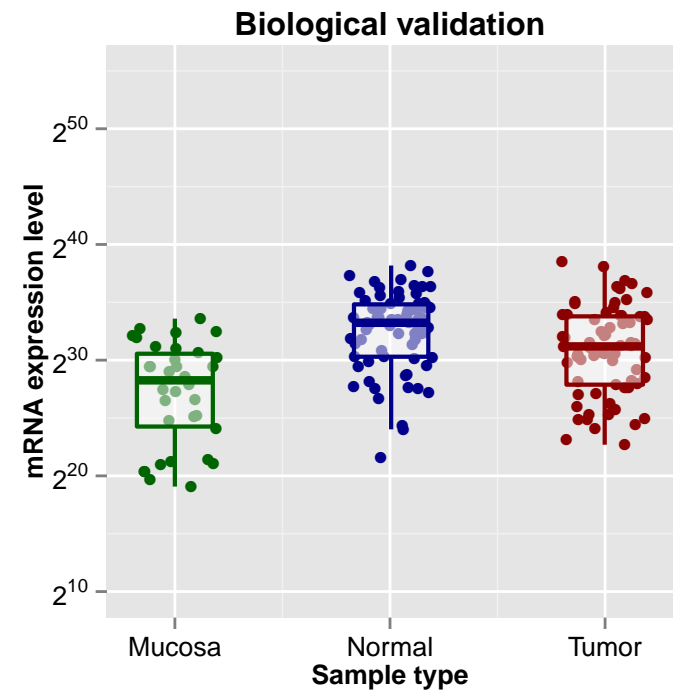

# THBS2

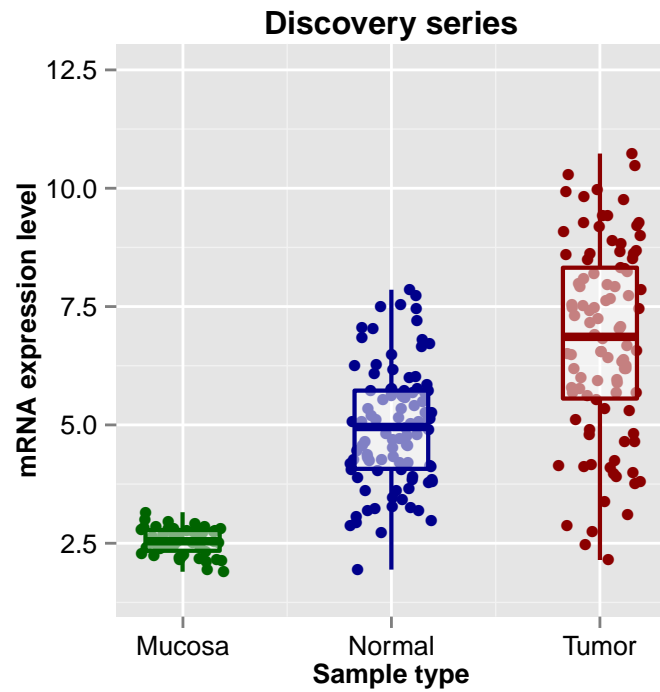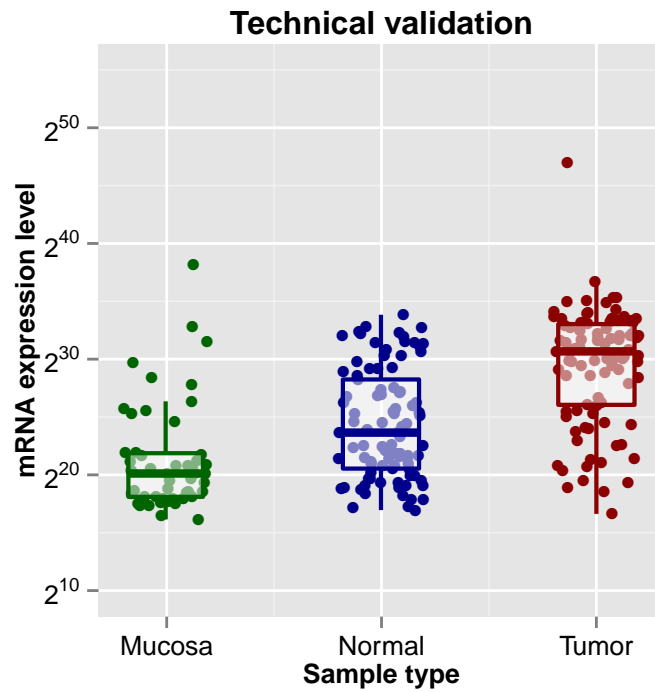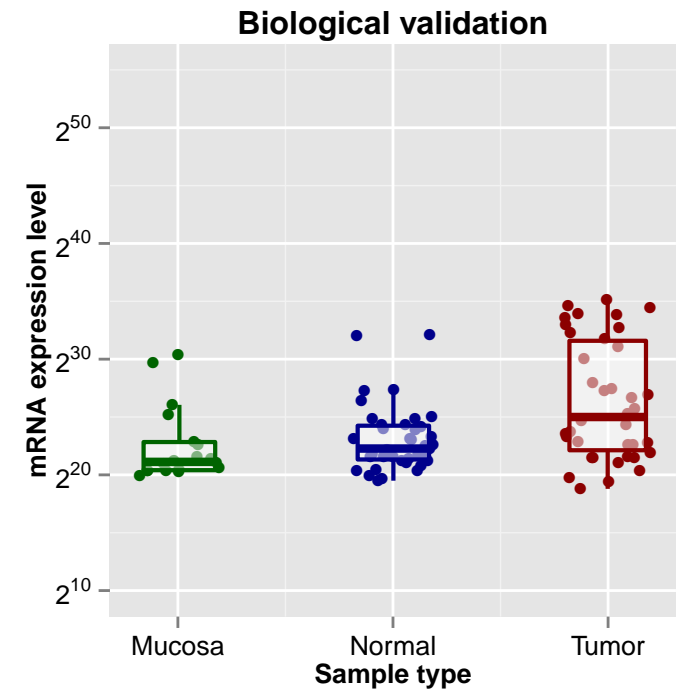

# TNC

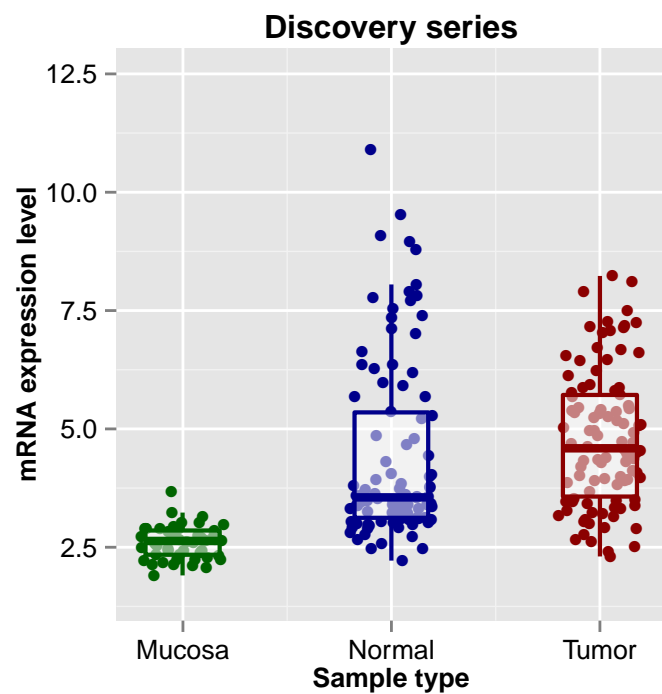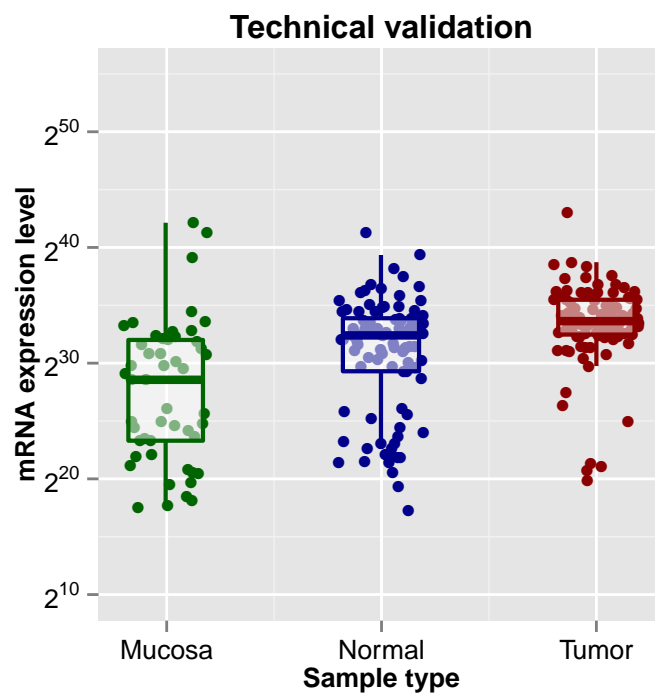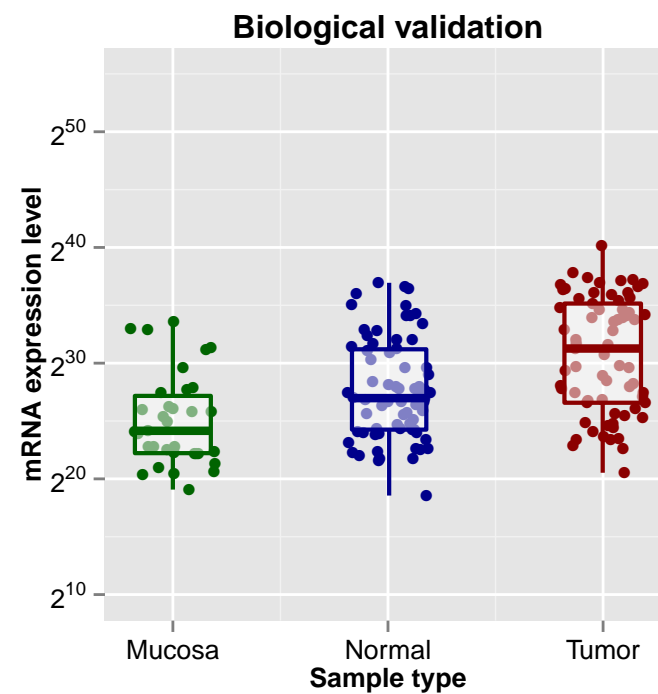

Supplement: Figure S3 — Expression levels for the discovery series, technical validation and biological validation of the 23 selected genes. (PDF) [file pone.0106748.s003.pdf]

# COL10A1

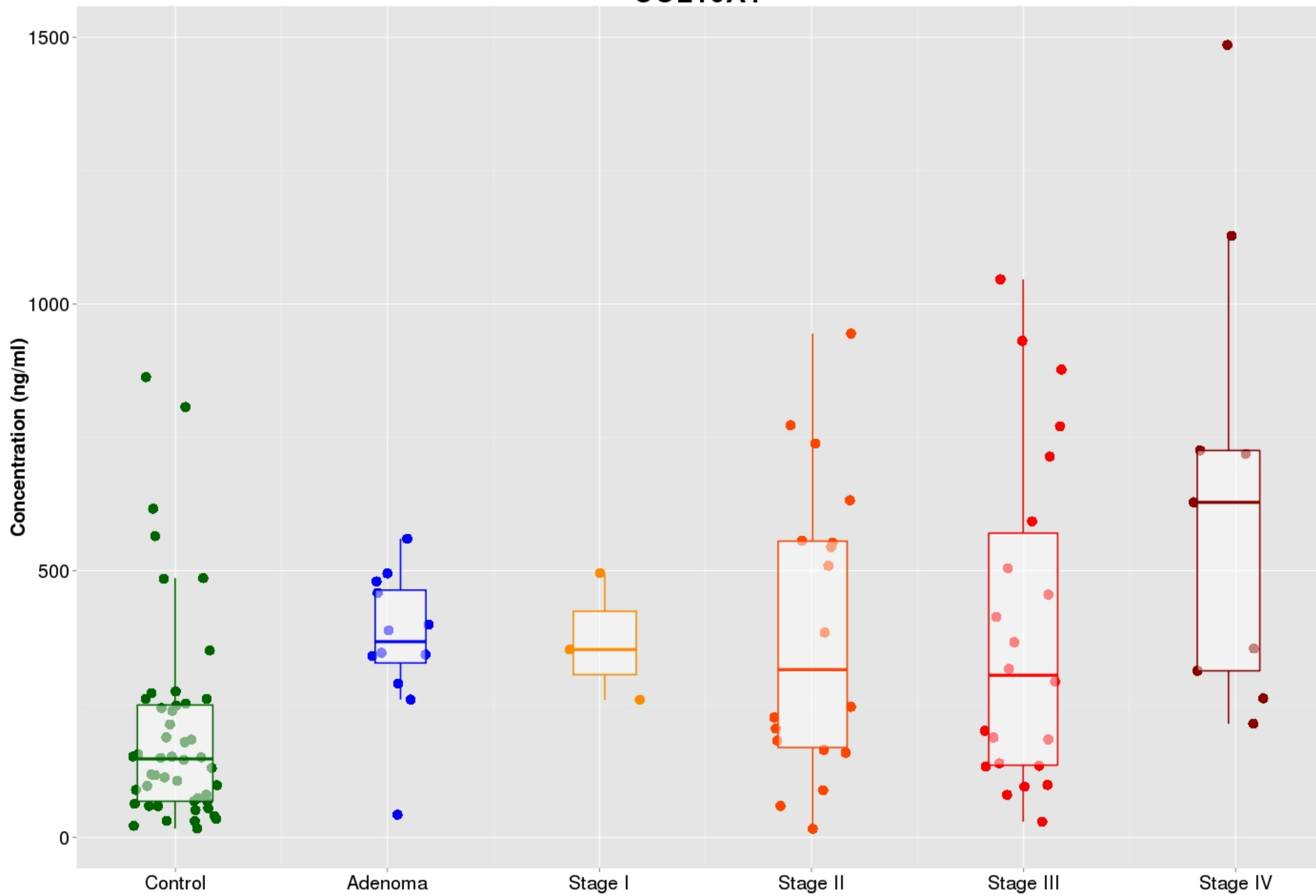

Supplement: Figure S4 — Serum concentration values of COL10A1 in relation to tumor stage. (PDF) [file pone.0106748.s004.pdf]

# COL10A1

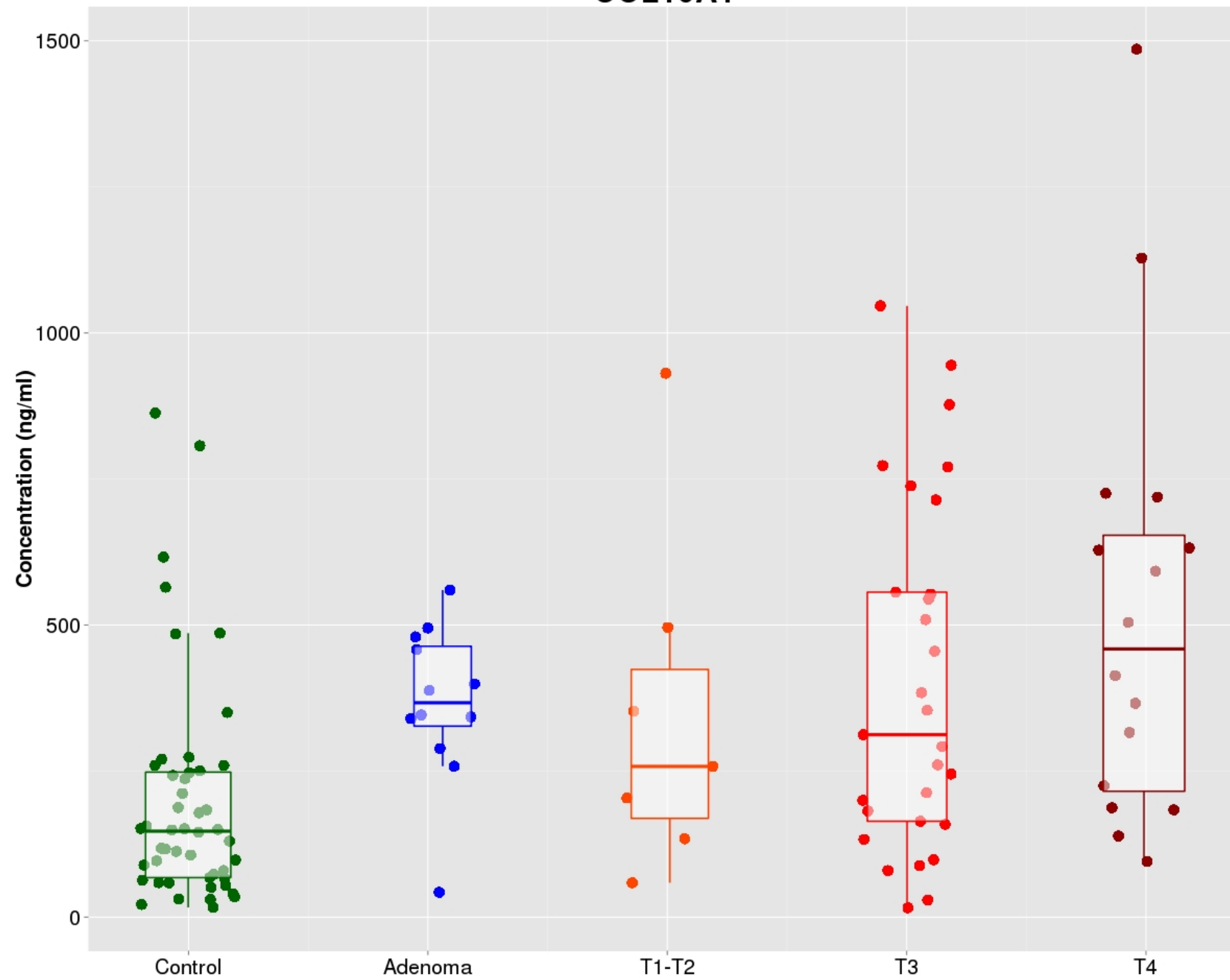

Supplement: Figure S5 — Serum concentration values of COL10A1 in relation to tumor size. (PDF) [file pone.0106748.s005.pdf]
