# Supplementary material for: Discovery and Validation of New Potential Biomarkers for Early Detection of Colon Cancer
Source: PLoS One. 2014 Sep 12;9(9):e106748. doi: 10.1371/journal.pone.0106748 (PMC4162553; doi:10.1371/journal.pone.0106748)
Supplement: Table S1 — Association of serum markers with patient characteristics, epidemiological factors and tumor characteristics. (DOCX) [file pone.0106748.s006.docx]

**Supplementary Table S1.** Association of serum markers with patient characteristics, epidemiological factors and tumor characteristics.

|  | ***Serum markers*** | | | | | | | | |
| --- | --- | --- | --- | --- | --- | --- | --- | --- | --- |
|  | **CEL** | **COL10A1** | **COL11A1** | **ESM1** | **GAL** | **KIAA1199** | **MMP10** | **MMP3** | **MMP7** |
| ***Patients characteristics*** |  |  |  |  |  |  |  |  |  |
| Age (p-value) | 0.27 | 0.26 | 0.29 | 0.24 | 0.35 | 0.32 | 0.49 | 0.12 | 0.010 |
| Gender (p-value) | 0.54 | 0.58 | 0.016 | 0.77 | 0.16 | **0.0039** | 0.15 | **1.6e-06** | 0.12 |
| ***Epidemiological factors*** |  |  |  |  |  |  |  |  |  |
| NSAIDs consumption (p-value) | 0.86 | 0.018 | 0.71 | 0.99 | 0.20 | 0.032 | 0.042 | 0.20 | 0.29 |
| BMI (p-value) | 0.71 | 0.44 | 0.46 | 0.38 | 0.20 | 0.82 | 0.59 | 0.24 | 0.25 |
| Alcohol consumption (p-value) | 0.18 | 0.29 | 0.30 | 0.68 | 0.80 | 0.19 | 0.50 | 0.15 | 0.86 |
| Tobacco consumption (p-value) | 0.60 | 0.039 | 0.31 | 0.016 | 0.036 | 0.35 | 0.32 | 0.078 | 0.31 |
| ***Tumor characteristics*** |  |  |  |  |  |  |  |  |  |
| Tumor localization (p-value) | 0.76 | 0.029 | 0.40 | 0.21 | 0.079 | 0.97 | 0.42 | 0.054 | 0.52 |
| Tumor stage (p-value) | 0.76 | 0.18 | 0.28 | 0.95 | 0.91 | 0.73 | 0.68 | 0.83 | 0.89 |
| Histological grade (p-value) | 1.0 | 0.84 | 0.80 | 0.52 | 0.14 | 0.14 | 0.32 | 0.39 | 0.22 |
| T - Primary tumor (p-value) | 0.031 | **0.00045** | 0.42 | 0.33 | 0.64 | 0.82 | 0.032 | 0.32 | 0.22 |
| Extramural vascular invasion (p-value) | 0.51 | 0.16 | 0.29 | 0.46 | 0.75 | 0.63 | 0.24 | 0.32 | 0.83 |

P-values derived from linear models. P-values < 0.01 are highlighted in bold.
